# Pupil Trend Reflects Suboptimal Alertness Maintenance over 10 s in Vigilance and Working Memory Performance: An Exploratory Study

**DOI:** 10.1523/ENEURO.0250-24.2024

**Published:** 2024-12-10

**Authors:** Jumpei Yamashita, Hiroki Terashima, Makoto Yoneya, Kazushi Maruya, Haruo Oishi, Takatsune Kumada

**Affiliations:** ^1^NTT Access Network Service Systems Laboratories, Nippon Telegraph and Telephone Corporation, Tokyo 180-8585, Japan; ^2^NTT Communication Science Laboratories, Nippon Telegraph and Telephone Corporation, Kanagawa 243-0198, Japan; ^3^Graduate School of Informatics, Kyoto University, Kyoto 606-8501, Japan

**Keywords:** alertness, attention network test, LC–NE, *n*-back, psychomotor vigilance, pupil

## Abstract

Maintaining concentration on demanding cognitive tasks, such as vigilance (VG) and working memory (WM) tasks, is crucial for successful task completion. Previous research suggests that internal concentration maintenance fluctuates, potentially declining to suboptimal states, which can influence trial-by-trial performance in these tasks. However, the timescale of such alertness maintenance, as indicated by slow changes in pupil diameter, has not been thoroughly investigated. This study explored whether “pupil trends”—which selectively signal suboptimal tonic alertness maintenance at various timescales—negatively correlate with trial-by-trial performance in VG and WM tasks. Using the psychomotor vigilance task (VG) and the visual–spatial two-back task (WM), we found that human pupil trends lasting over 10 s were significantly higher in trials with longer reaction times, indicating poorer performance, compared with shorter reaction time trials, which indicated better performance. The attention network test further validated that these slow trends reflect suboptimal states related to (tonic) alertness maintenance rather than suboptimal performance specific to VG and WM tasks, which is more associated with (phasic) responses to instantaneous interference. These findings highlight the potential role of detecting and compensating for nonoptimal states in VG and WM performance, significantly beyond the 10 s timescale. Additionally, the findings suggest the possibility of estimating human concentration during various visual tasks, even when rapid pupil changes occur due to luminance fluctuations.

## Significance Statement

Using biomarkers to estimate human concentration levels can adaptively enhance performance in daily activities. Theoretically, the pupil diameter, which measurably fluctuates over several seconds, could mirror real-time concentration in demanding tasks like vigilance (VG) and working memory (WM). Although capable of accurately estimating concentration in the presence of rapid luminance changes, empirical evidence linking these pupil measures at the slow timescales to trial-by-trial VG and WM task performance is lacking. This study demonstrates that the 10 s pupil trend accurately reflects these tasks’ performance, underscoring its potential for daily concentration assessment.

## Introduction

Success in maintaining concentration for several seconds varies, with failures potentially leading to significant consequences for everyday activities. Cognitive psychology identifies vigilance (VG) and working memory (WM) tasks as requiring such concentration, with both showing trial-by-trial performance variations (DeBettencourt et al., 2019). A VG task is an attention task that demands readiness to respond to unpredictable stimuli within a 2–10 s latency period, exemplified by the psychomotor vigilance task (PVT; Wilkinson and Houghton, 1982; Loh et al., 2004). WM tasks involve retaining a stimulus while processing another stimulus, requiring retrieval after a period exceeding 5 s, such as in the two-back task (2BT; Cohen et al., 1997; Carlson et al., 1998). The maximum variable latency period of up to 10 s is a critical commonality between VG and WM tasks (Unsworth et al., 2020; Unsworth and Robison, 2020). Therefore, fluctuations in internal state maintenance at relatively long timescales (e.g., 10 s) may explain trial-by-trial performance variations in these tasks.

Neuroscience studies have suggested that such variations in internal state maintenance may be reflected in slow pupil changes (Table 1; Aston-Jones and Cohen, 2005; Unsworth and Robison, 2017). The neural networks commonly active during VG and WM tasks—such as the thalamus and the anterior insula and frontal operculum (AI/FO; Rottschy et al., 2012; Langner and Eickhoff, 2013)—slowly detect and signal the need to internally maintain response preparation for unpredictably timed targets (i.e., “tonic” alertness maintenance; Sturm and Willmes, 2001; Sterzer and Kleinschmidt, 2010; Coste and Kleinschmidt, 2016). The AI/FO activity increases with insufficient alertness (poorer performance) as the need for maintenance increases due to persistent adverse effects (Sadaghiani and D'Esposito, 2015). Through the locus ceruleus–norepinephrine (LC–NE) system connected to pupil diameter regulation, signals of internal maintenance inefficiency common to VG and WM tasks would manifest in slow pupil changes, which negatively correlate with trial-by-trial task performance (Aston-Jones and Cohen, 2005; Gilzenrat et al., 2010; Medford and Critchley, 2010; Ullsperger et al., 2010; Joshi et al., 2016; Schneider et al., 2016). These slow responses contrast with the fast AI/FO (pupil) response to external interference events, which deviates from the preceding preparatory state, thereby indicating improved event detection (i.e., “phasic” alertness response positively correlated with performance; Corbetta and Shulman, 2002; Menon and Uddin, 2010; Sterzer and Kleinschmidt, 2010; Ullsperger et al., 2010; Tian et al., 2014; Harsay et al., 2018).

**Table 1.** Background summary of tonic and phasic signals in VG and WM tasks

	Tonic maintenance signal	Phasic response signal
Meaning	Alertness maintenance at the (increased) level required by persistent adverse factors	Alertness response to external instantaneous deviation (e.g., salience or interference) onset
Timescale	Long	Short
Correlation with performance	Negative	Positive

Despite these backgrounds, pupillometric studies have left a gap in evidence for tonic pupil signals negatively related to trial-by-trial VG and WM performance. The “baseline (pretrial) pupil size” is thought to reflect internal states present before the start of a trial, potentially influencing responses after the variable latency period (e.g., 2–10 s) or retention period (e.g., 5 s; Unsworth and Robison, 2018; Robison and Unsworth, 2019). However, findings regarding the relationship between the baseline pupil size and task performance have been inconsistent. Studies have consistently shown a negative correlation between baseline variability and performance (Unsworth and Robison, 2017; Unsworth and Miller, 2021), but it remains unclear whether increases or decreases in the baseline size are more closely linked to performance outcomes (Martin et al., 2022). This inconsistency likely arises from the wide range of timescales involved in the baseline pupil size, which may obscure the specific role of tonic pupil signals over long timescales that would negatively associate with performance (Van Den Brink et al., 2016).

To address these inconsistencies, this study introduces “pupil trends” as novel indices that capture slow, sustained changes in pupil diameter and explores long-timescale changes (e.g., 10 s) that negatively correlate with VG and WM performance. Inspired by previous temporal analyses of pupil changes (Yamashita et al., 2021, 2022), we isolate pupil diameter changes at specific timescales using smoothing methods. This procedure filters out fast temporal components influenced by rapid luminance fluctuations in tasks or phasic responses.

We examined the relationship between pupil trends with different temporal resolutions and trial-by-trial performance in PVT (i.e., VG) and 2BT (i.e., WM) tasks (Wilkinson and Houghton, 1982; Cohen et al., 1997; Carlson et al., 1998; Loh et al., 2004). Furthermore, we employed the attention network test (ANT; Fan et al., 2005) to demonstrate that pupil trends signal the need for maintaining tonic alertness rather than the phasic response to external interference. Since pupil responses to changes in stimulus luminance are typically immediate, finding evidence that signals related to human concentration persist over these slow timescales could represent an essential step toward estimating concentration during complex cognitive tasks.

This study builds upon data we collected in our earlier research (Yamashita et al., 2021), where we presented findings from PVT data on the micro-pupillary unrest index (Yamashita et al., 2021, 2022). The data underlying this study's pupil trend differ from the analyses in the previous study. We have yet to publish reports on the 2BT and ANT data.

## Materials and Methods

### Participants

Part-time job applicants (*N* = 20, 15 females; age range, 20 and 43 years) with normal or corrected-to-normal vision participated in this study. The Nippon Telegraph and Telephone (NTT) Communication Science Laboratory Research Ethics Committee approved all experimental procedures (Reference Number H29-004). A written informed consent was obtained from every participant. They were recruited externally and compensated for participating.

### Apparatus and stimuli

Participants were seated 60 cm from a 27 inch LCD monitor with a 144 Hz refresh rate and 1,920 × 1,080 resolution. They engaged with stimuli presented on a black background via a Python (2.7.14) and Psychopy2 (Peirce, 2007, 2009) environment controlled by a desktop computer (Windows 7). Binocular eye movements were recorded at 1,000 Hz using the SR Research EyeLink 1000, with participants’ heads stabilized by a chin rest throughout the tasks.

### Procedures

We aimed to provide convergent evidence that slow pupil signals indicative of insufficient (suboptimal) tonic alertness potentially may underlie the shared trial-by-trial performance decrements in VG and WM tasks. Participants performed eight tasks, with three selected for analysis in this study [i.e., VG task (PVT), WM task (2BT), and ANT]. The task order was randomized, and each task lasted ∼10 min. Sessions were designed such that two participants alternated between performing tasks and taking breaks, with the entire procedure spanning 180–210 min, including the preparations.

#### Tasks

We employed a VG task (i.e., PVT), which is associated with the tonic alertness concept, and a WM task (i.e., 2BT), which corresponds to fast phasic interference detection and resolution, to gather substantial evidence for the tonic alertness's relevance across both domains (compare Table 1). In general, VG and WM tasks require participants to maintain a preparatory state to counteract nonoptimality during the latency (VG) or retention (WM) periods (tonic alertness maintenance; Posner and Boies, 1971; Posner, 1978; Rottschy et al., 2012; Langner and Eickhoff, 2013). At the same time, participants must also manage interference by identifying external targets amidst competing internal states, such as mind wandering (VG) or concurrent interfering memories (WM; phasic alertness response; Sridharan et al., 2008; Burgess and Braver, 2010; Levens and Phelps, 2010; Langner and Eickhoff, 2013; Unsworth and Robison, 2016). To separately attribute these aspects to VG and WM tasks, we employed the PVT (Wilkinson and Houghton, 1982; Loh et al., 2004) and the 2BT (Cohen et al., 1997; Carlson et al., 1998). If pupil trends consistently show correlations, it would underscore the tonic alertness's role across these domains.

We selected the PVT-based simple reaction task described by Mueller and Piper (2014) and Wilkinson and Houghton (1982) for the VG task. The PVT has been widely utilized to explore the overlap in cognitive demands between VG and WM tasks (Unsworth and Robison, 2017; Unsworth et al., 2020; Unsworth and Miller, 2021). The PVT demands a swift response to targets after a variable latency period, necessitating participants to sustain their response preparation throughout this interval (tonic alertness maintenance; Posner and Boies, 1971; Posner, 1978; Langner and Eickhoff, 2013). This task contrasts with other VG tasks, such as the sustained attention to response or the continuous performance task. These alternative tasks could complicate the execution of specific reactions due to the repetitive nature of other required responses, a phenomenon known as interference detection/resolution (Rosvold et al., 1956; Robertson et al., 1997).

For the WM task, we selected the 2BT, following the methodology of a prior study (Carlson et al., 1998). In the 2BT (WM task), the participants perform the interfering task of remembering the stimulus presented in trial *n* for retrieval in trial *n* + 2 while remembering the stimulus presented in trial *n* + 1 for retrieval in another trial *n* + 3. This task challenges participants to correctly detect and resolve the two interfering memory tasks, requiring continuous detection and resolution of interference (phasic alertness response; Burgess and Braver, 2010; Levens and Phelps, 2010). This demand for explicit detection and resolution of interference distinguishes the 2BT from tasks like the whole report procedure, which assesses memory capacity without directly addressing interference (Adam et al., 2015).

To examine the relationship between the pupil trend and individual performance in VG and WM tasks, we utilized reaction time (RT) as a differential marker of performance quality. An uptrend was predicted to correspond with poorer performance (longer RTs). RT in the PVT serves as a reliable VG performance indicator, varying from trial to trial (Unsworth et al., 2018). While WM performance in the 2BT is often gauged by accuracy (Haatveit et al., 2010), RT offered an alternative metric due to the scarcity of incorrect responses, thus providing a broader base for pupil index analysis. RT, within this context, is considered a proxy for WM performance (Jacola et al., 2014).

Furthermore, utilizing the ANT (Fan et al., 2005) allowed us to examine whether the trend index signifies the need for tonic alertness maintenance for unpredictably timed targets rather than immediate external interference resolution (phasic alertness response). The ANT's alerting comparison could differentiate between conditions with and without predictive cues for target timing. This suggests that an absence of cues (no-cue condition) would elevate the slow pupil trend relative to the presence of cues (Sterzer and Kleinschmidt, 2010; Sadaghiani and D'Esposito, 2015; Coste and Kleinschmidt, 2016). Conversely, the executive control comparison, contrasting conditions with direct interference against those without (Ullsperger et al., 2010; Laeng et al., 2011; Geva et al., 2013), expected no significant variation in the pupil trend.

##### PVT (VG)

**Figure 1.**
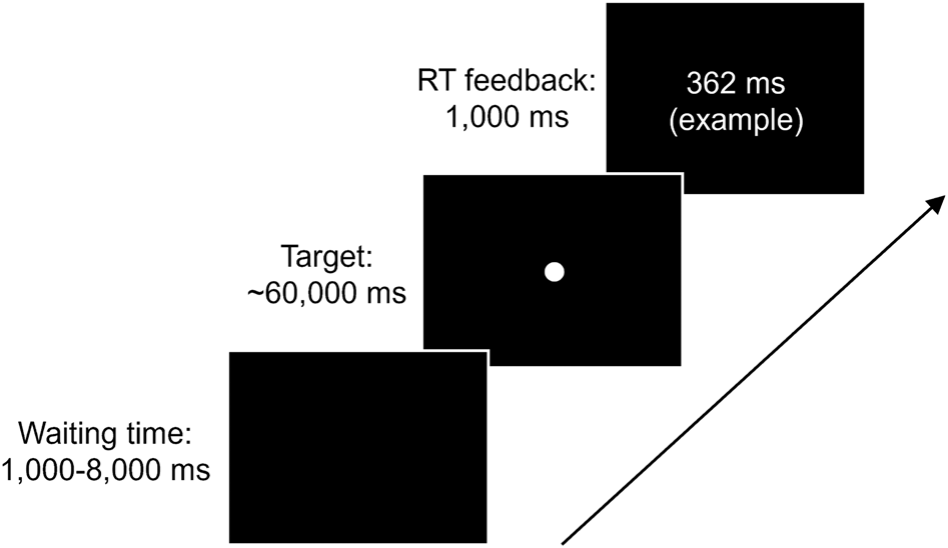
Sequence of elements in a PVT trial.

Participants were tasked with reacting to a target following a variable latency period ranging from 1,000 to 8,000 ms in 250 ms steps by pressing the “space” key as swiftly as possible (Fig. 1). This 1–8 s latency span bridges the standard period of 2–10 s (Loh et al., 2004) and the shorter 1–4 s variant (Basner et al., 2011). The target, a white circle with a visual angle of 2.90°, appeared at the screen's center. Each participant performed 29 latency periods, each consisting of four trials. Upon pressing the “space” key, the reaction time was shown for 1,000 ms. A “False Alarm!” alert was displayed if a participant responded before the target appeared. Conversely, failing to respond within 60 s triggered a “Miss!” alert. Following the disappearance of these alerts, the subsequent trial commenced immediately.

##### 2BT (WM)

**Figure 2.**
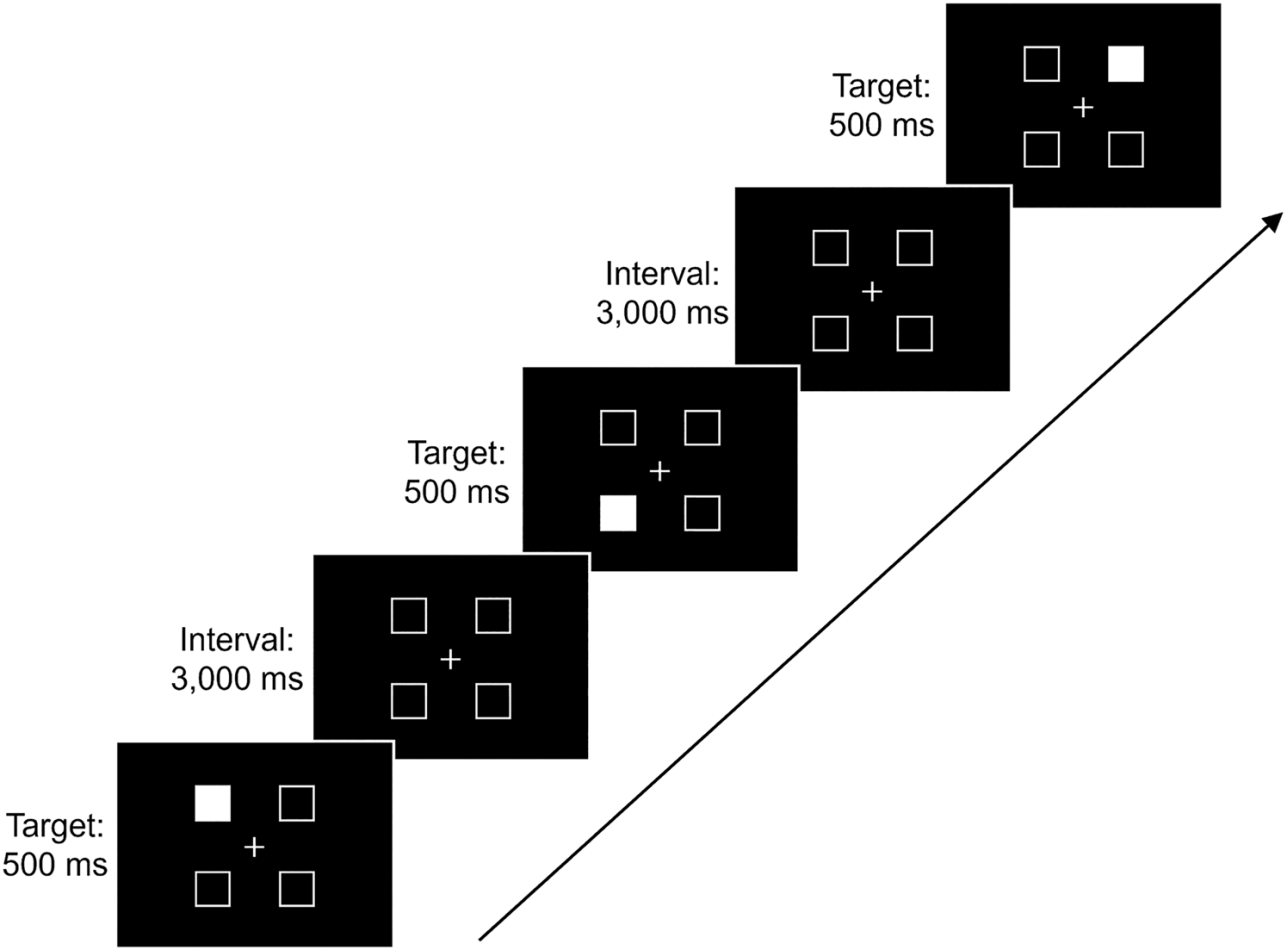
Sequence of elements in a 2BT response.

In the 2BT, participants determined if the current target matched the position of the one presented two trials earlier. They responded by pressing the “right” (for “same”) or “left” (for “different”) key as promptly as possible (refer to Fig. 2). A fixation point remained constant at the screen's center, flanked by four rectangular frames. Each trial involved one frame turning white (indicating the target) for 500 ms before reverting to black for 3,000 ms. The rectangular frames set 1.48 visual degrees in size were positioned 3.80° from the screen's center horizontally and vertically. Initially, each of the four positions had an equal (25%) chance of being the target. Subsequently, target selection was based on a 50% chance from locations used in the two preceding trials, combined with the remaining positions. A nontarget position from the previous trials was chosen with a 33% probability for the latter. Participants encountered 160 targets across 158 trials (cf., no response for the first two targets).

##### ANT

**Figure 3.**
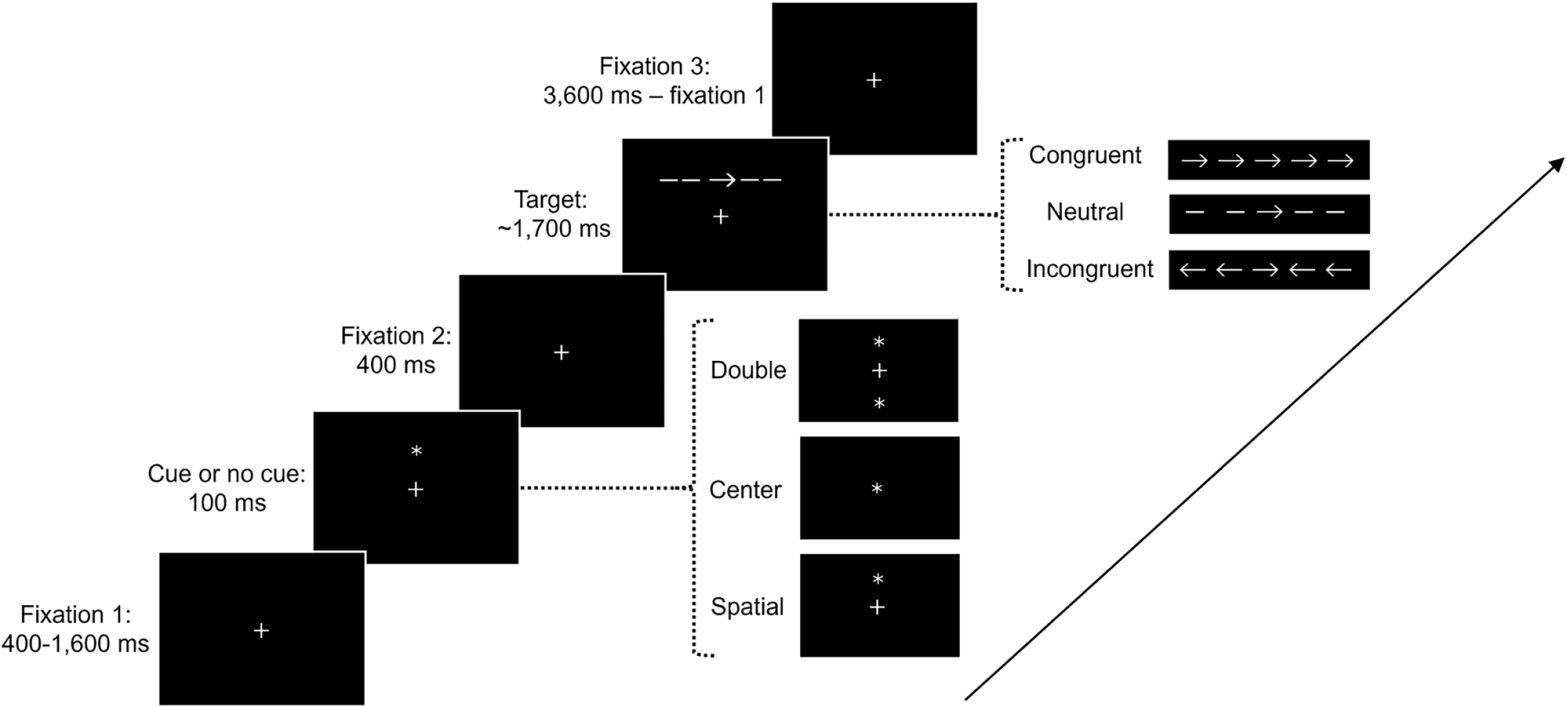
ANT trial sequence. This figure details the sequence of cue presentations in an ANT trial. The “no-cue” condition shows only the fixation cross. The “double-cue” condition presents two asterisks, indicating both potential target positions (above and below). The “center-cue” condition features an asterisk at the fixation cross's location, while the “spatial-cue” condition places an asterisk at the actual target position (above or below) as a valid predictor of the target's location. The appearance of each cue type is randomized, occurring with equal probability (25%) across trials. The three kinds of target stimuli were as follows: the congruent target stimuli consisted of five arrows pointing in the same direction; the neutral target stimuli consisted of a central arrow and four flankers of line segments; the incongruent target stimuli consisted of a central arrow and four flankers of arrows pointing in opposite directions.

In the ANT, participants determined the direction (right or left) of the central arrow in target stimuli by pressing the “right” or “left” keys swiftly on the keyboard (Fig. 3). The task began with a fixation period of random length (400–1,600 ms), followed by a 100 ms cue presentation. Cue types included no cue, double cue, center cue, or spatial cue, succeeded by a 400 ms fixation before target appearance. Targets could be congruent, neutral, or incongruent, featuring a central arrow flanked by four others, positioned above or below the fixation point with equal probability (50%) on a trial-by-trial basis. The direction of the central arrow varied randomly (50% chance). Target stimuli, measuring 3.08 visual degrees with each arrow or line spanning 0.55° and a 0.06° separation between adjacent elements, remained visible until a response was made or for a maximum of 1,700 ms. Following a response, targets vanished, leading to a variable post-target fixation period (3,600 ms minus the initial fixation duration). Participants completed 96 trials in total. The alerting comparison contrasted the center and no cues, regardless of target type [we did not compare the difference between no cue and double cue as the alerting comparison, rigorously addressing the possibility of luminance difference between double cue and no cue affecting the biological signal (here, pupil signal), following previous studies (Fan et al., 2005). Also, this study did not include the comparison of orienting effects based on spatial vs center cues]. Conversely, the executive control comparison assessed performance differences between congruent and incongruent targets, irrespective of cue type, to evaluate conflict resolution capabilities.

### Pupil measurement

We measured task-independent pupil changes within a 5–15 s interval as universal indices of pupil temporal components. We employed a smoothing technique to calculate this “smoothed trend,” demonstrating its significant effect size with straightforward methods. We also utilized multiresolution decomposition to determine which timescale changes in pupil trends were significant (i.e., “decomposed trend”). Additionally, we presented indicators for changes occurring within intervals of <5 s and >15 s, which were not the subject of statistical tests.

#### Pupil trend calculation

**Figure 4.**
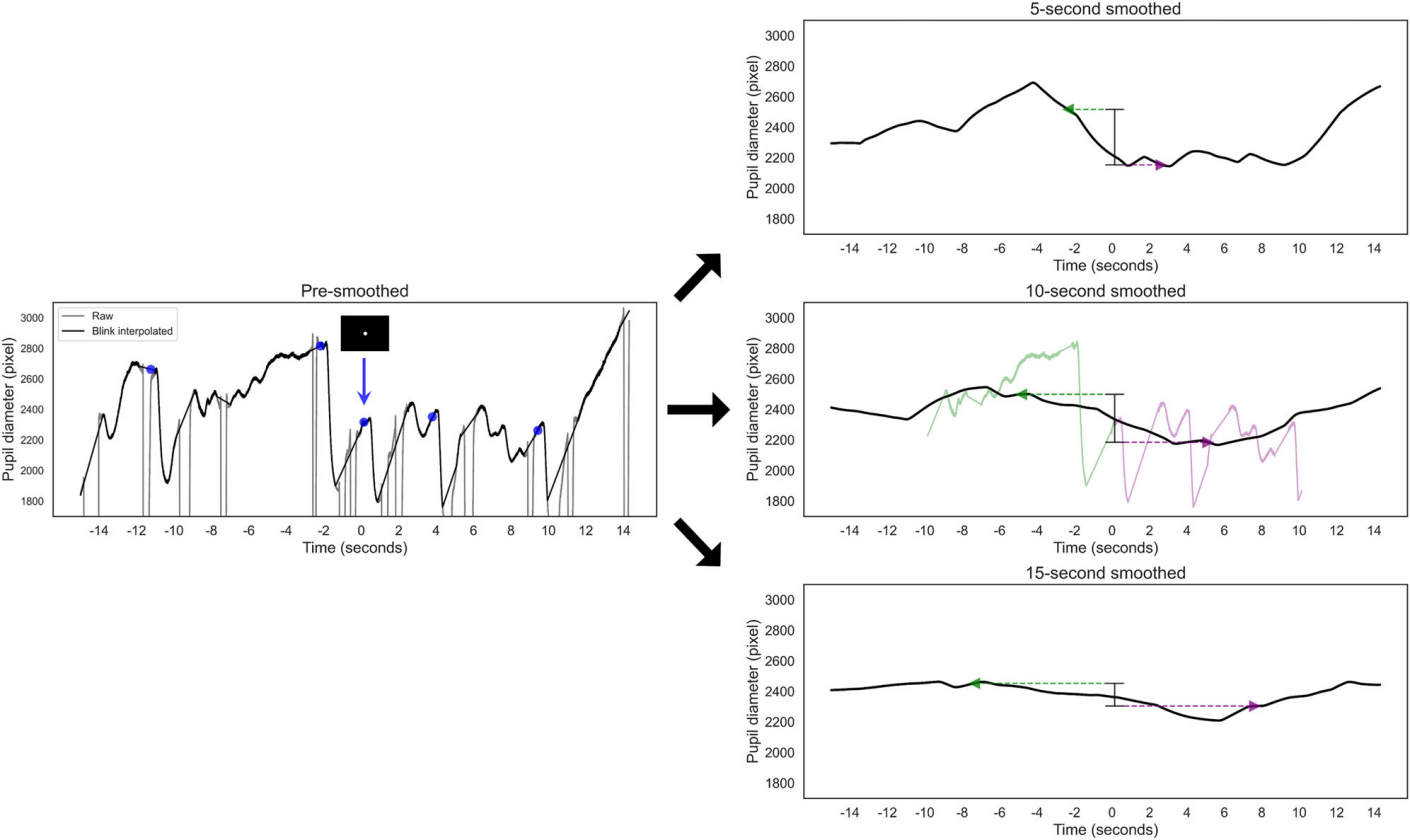
Conceptual illustration of smoothed trend calculation. This figure illustrates the process of calculating the smoothed trend within a PVT. The left panel displays the raw pupil diameter (gray line), including blinks, and the blink interpolation (black line). The presmoothed pupil diameter rapidly constricts when the white target appears, as indicated by the blue marker. In contrast, the right panels show pupil diameters smoothed over 5–15 s windows, highlighting the method's sensitivity to slower pupil changes unaffected by rapid shifts in luminance. The trend calculation reflects the pupil's expansion or contraction over the specified time window, centered on the moment of the target display. This is calculated by subtracting the diameter indicated by the green marker from that indicated by the purple marker. In the case of 10 s smoothing, this corresponds to subtracting the mean presmoothed diameters shown in the light green graph from those in the light purple graph.

**Figure 5.**
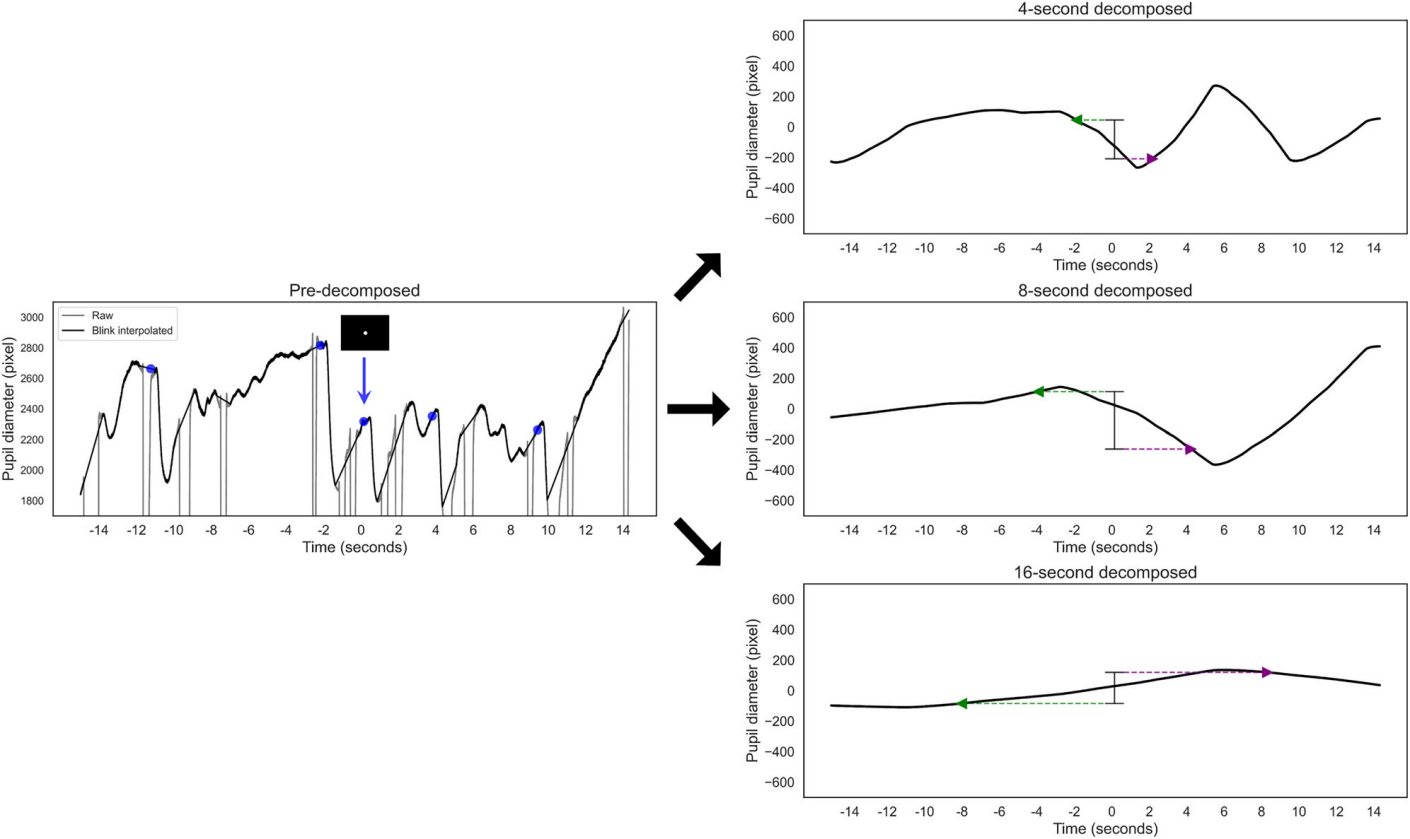
Conceptual illustration of decomposed trend calculation. This figure illustrates the calculation process for the decomposed trend within a PVT. The left panel shows the raw and blink-interpolated pupil diameters as gray and black lines, respectively. These diameters rapidly constrict when the white target appears, as indicated by the blue marker. In contrast, the right panels display the decomposed pupil diameter, representing the fluctuating component with a time resolution of 4–16 s, which is unaffected by rapid luminance shifts. In each decomposition, the trend calculation subtracts the decomposed diameter indicated by the green marker from that indicated by the purple marker.

Preprocessing began with the exclusion of time series data related to pupilar diameter during eye closures (Figs. 4, 5, left panels). A noise-robust method identified blinking times (Hershman et al., 2018), with 200 ms before and after detected blinks excluded to avoid artifacts. Missing pupil diameter data were interpolated linearly.

The pupil trend, defined as the change in pupil diameter centered at the target presentation across all tasks and trials, was then calculated (Figs. 4, 5, right panels). This calculation involved transforming the preprocessed pupil diameter data into trend components through smoothing or multiresolution decomposition. The changes in these smoothed or decomposed diameters were calculated by averaging the indices for both eyes to account for binocular measurements.

##### Smoothed trend

For smoothing, we initially processed the pupil diameter time series using smoothing windows of 5, 10, and 15 s (Fig. 4). This procedure involved calculating average values across these windows as they moved through the blink-interpolated pupil diameter data. Subsequently, trend indices were derived from the changes in pupil diameter over the respective window durations.

For example, with a 10 s window, the change in pupil diameter over this period was quantified by measuring the increase or decrease. The change was calculated by subtracting the smoothed pupil diameter at the beginning of the 10 s window from that at the end. The value at the start represents the smoothed pupil diameter 5 s before the target presentation (i.e., the average of the presmoothed pupil diameter from −10 to 0 s), and the value at the end represents the smoothed pupil diameter 5 s after the target presentation (i.e., the average of the presmoothed pupil diameter from 0 to 10 s).

As a supplementary measure, we used the “conservative trend,” which calculates changes over 14 s intervals while using a 10 s window for smoothing. In this approach, the initial smoothed value is the pupil diameter 7 s before the target presentation (i.e., the mean of the raw pupil diameter from −12 to −2 s), and the final smoothed value is the pupil diameter 7 s after the target presentation (i.e., the mean of the raw pupil diameter from 2 to 12 s). This conservative analysis was designed to carefully exclude the effects of phasic activity that might occur within a 4 s range around the target presentation.

##### Decomposed trend

In the multiresolution decomposition approach, we segmented the pupil diameter time series data into components with varying temporal resolutions (Fig. 5). This segmentation was accomplished using a discrete wavelet transform, specifically the Symlet-4 wavelet (Lee et al., 2019). This technique allowed for scalings at resolutions of 2^n^, including 66 (65,536 ms), 33 (32,768 ms), 16 (16,384 ms), 8 (8,192 ms), 4 (4,096 ms), down to 0.002 (2 ms) seconds.

Each scaling level, starting from the 66 s scale, contained data on the pupil diameter value at that specific temporal scale. Signals at the 33 s scale and below were distinct, focusing solely on the magnitude of changes at those finer resolutions, isolated from broader-scale fluctuations. This procedure enabled us to calculate pupil diameter variance precisely by analyzing the data obtained from each distinct temporal scale, specifically at the 33, 16, 8, and 4 s resolutions.

For instance, at an 8 s resolution, the change in pupil diameter over this period was quantified. By examining the time series data at an 8 s temporal resolution, we compared the pupil diameters 4 s before and after the target presentation. The trend was calculated by subtracting the earlier value from the later one.

### Code availability

The data analysis code/software is freely available online at https://osf.io/3b5fv (Yamashita et al., 2024). Researchers can use the code under a license set by NTT, which allows them to use it for scientific evaluation. The data analysis was performed in a Python (3.8.17) environment controlled by a laptop computer (Windows 10, Intel Core i5-8265U CPU, 8GB RAM).

## Results

In the data analysis, we first presented the trial-by-trial differences in pupil trends between varying VG and WM performances in a straightforward manner (i.e., preliminary analysis) before examining which specific temporal resolutions of the trends differed between distinctly different performance levels (i.e., primary analysis).

### Descriptive statistics

#### PVT (VG)

Valid pupil data were provided by 18 participants after excluding one due to technical issues and another for having their eyelids partially closed throughout the task. These participants conducted a total of 2,088 trials. They accurately responded to most trials, with no missed trials and only nine false alarms, including anticipatory reactions with RTs of 150 ms or less. Erroneous trials were excluded from the analysis. The initial 5% (six trials) were also removed to eliminate potential noise from task initiation, such as pupil diameter adjustments to darkness, leaving 1,971 of the 2,088 trials valid. The average RT for each participant for these trials was 390.31 ms, with an average standard deviation (SD) of 87.94 ms.

#### 2BT (WM)

Participants (*N* = 17) contributed valid pupil data after excluding one for technical errors, another for consistent partial eyelid closure, and a third for uncorrected vision during the task because the participants forgot to wear their eyeglasses. They completed a total of 2,686 trials. Of these, 2,435 trials were answered correctly, with 239 incorrect answers, 12 instances of no response, and no premature reactions under 150 ms. Incorrect and nonresponses were excluded. Additionally, the first 5% (eight trials) were omitted, resulting in 2,313 valid trials out of 2,686. The average RT for each participant was 1,098.15 ms, with an average SD of 376.67 ms.

#### ANT

The 17 participants yielded valid pupil data after excluding one for technical errors and two whose eyelids were half-closed throughout the task. These participants completed 1,632 trials, accurately responding to 1,551 trials, with 58 incorrect responses, 23 instances of no reaction, and no premature responses within 150 ms or less. Excluding erroneous trials and the initial 5% for valid conditions (three trials) for potential startup noise, 1,503 out of 1,632 trials were deemed valid. Each participant's average RT was 673.72 ms, with an average SD of 119.99 ms.

### Preliminary analysis

We first examined the correlation between the smoothed pupil trend and performance timing in PVT (VG) and 2BT (WM), categorizing trials with RTs below the participant's average as good performance and those with longer RTs as poor. An analysis was conducted to determine if average pupil trends varied between these performance levels in both tasks after excluding a few trials at the start and the end without valid pupil trend data due to smoothing window constraints. We also used the “conservative trend” to highlight trend differences while excluding pupil changes around the target presentation. Additionally, we conducted supplementary analyses comparing correct and incorrect responses in the 2BT.

For PVT (VG), the average RT for short response trials was 344.52 ms (SD, 32.69), while long response trials averaged 471.67 ms (SD, 89.68; *t*_(17)_ = −7.67; *d *= −1.83; *p *< 0.001). In 2BT (WM), short RTs averaged 869.72 ms (SD, 162.08), with long RTs at 1,490.30 ms (SD, 321.07; *t*_(16)_ = −13.50; *d *= −2.37; *p *< 0.001). Additionally, the average RT for correct responses in 2BT was 1,098.15 ms (SD, 222.05) and for incorrect responses is 1,439.05 ms (SD, 304.59; *t*_(16)_ = −6.80; *d *= −1.24; *p *< 0.001).

**Figure 6.**
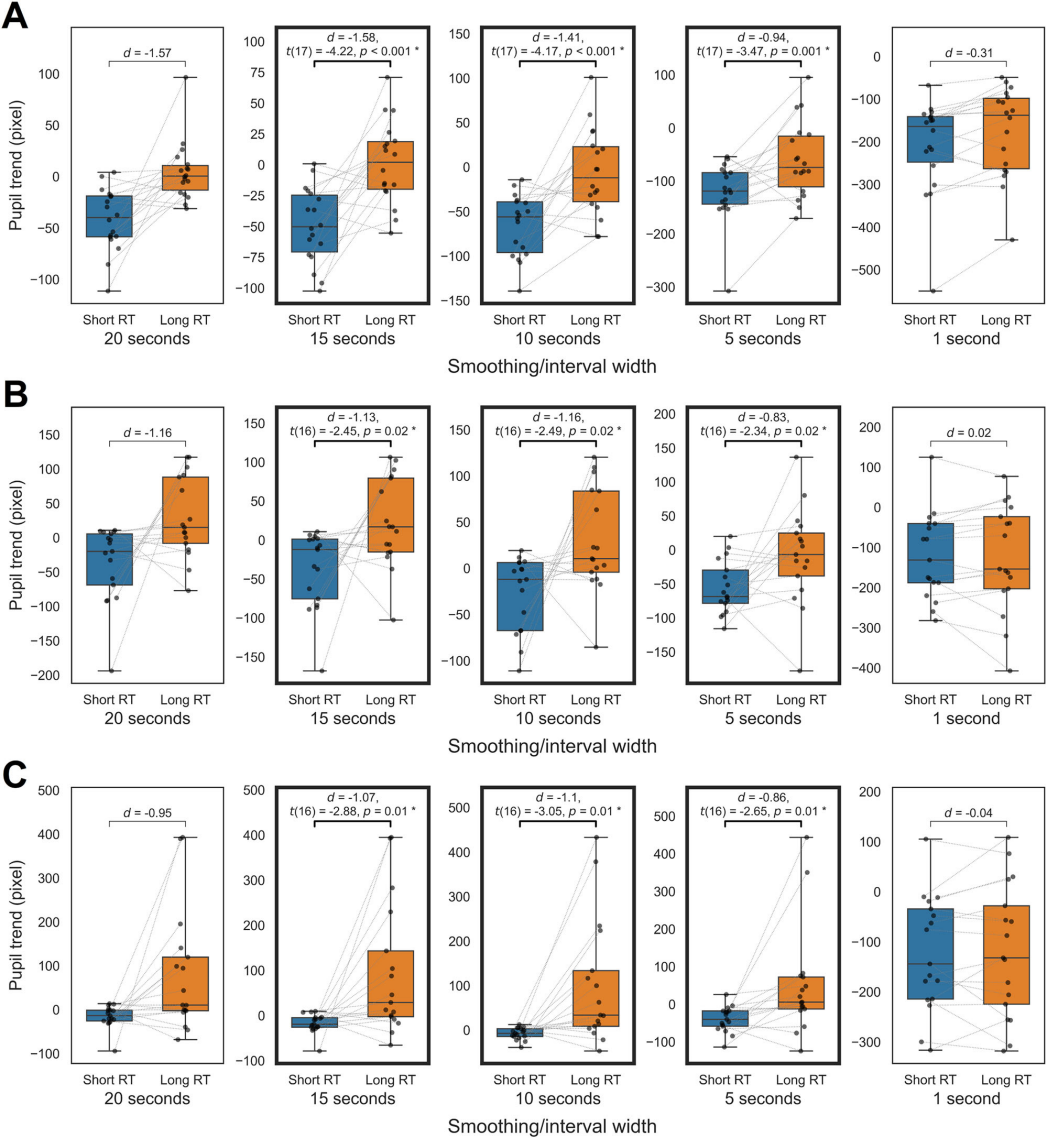
Preliminary pupil comparison using the smoothing method. ***A***, Short and long RT in PVT (VG). ***B***, Short and long RT in 2BT (WM). ***C***, Correct and incorrect responses in 2BT (WM).

The initial analysis focused on pupil trends derived from moving averages, which revealed relatively large effect sizes for windows above 10 s. For PVT, the analysis of smoothed trends from various smoothing windows (Fig. 6*a*) revealed that trends from windows above 5 s were significantly lower in short RT trials than long ones [*p *< 0.05; false discovery rate (FDR) corrected, one-tailed]. Similarly, in 2BT (Fig. 6*b*), trends from windows over 5 s showed significantly lower values in short RT trials than in long ones (*p *< 0.05; FDR corrected). Also, the 10 s conservative trends were lower in short RT trials than long ones (VG, *t*_(17)_ = −3.67; *d *= −1.53; *p *< 0.001; WM, *t*_(16)_ = −2.53; *d *= −1.17; *p *= 0.011). Additionally, a supplementary comparison of 2BT correct and incorrect responses (Fig. 6*c*) indicated that trends over 5 s windows were significantly lower in correct trials compared with incorrect ones (*p *< 0.05; FDR corrected). Although we did not develop a precise transformation formula, it is worth noting that the 10 s trend differences (59.23 pixels in the PVT and 56.61 pixels in the 2BT) were ∼0.07–0.08 mm. This estimation is based on the assumption that the mean pupil size at the PVT target presentation of 3,300 pixels corresponds to a generally observed pupil size of 4.5 mm against a dark background in a dimly lit room (Peysakhovich et al., 2015).

### Primary analysis

We conducted a rigorous investigation to determine which temporal resolutions of the trends differed between markedly different performance levels.

#### PVT (VG) and 2BT (WM)

We examined trend differences in PVT (VG) and 2BT (WM) by categorizing trials with RTs below the participant's 25th percentile as indicative of solid performance and those above the 75th percentile as indicative of poor performance. We first conducted the analysis using smoothed trends to ensure consistency with the preliminary analysis. We then applied decomposed trends to identify significant changes across different timescales.

For PVT (VG), the average RT for the fastest 25% of trials was 319.57 ms (SD, 28.01), while the slowest 75% averaged 498.26 ms (SD, 97.16; *t*_(17)_ = −8.57; *d *= −2.43; *p *< 0.001). In 2BT (WM), the fastest 25% of short RTs averaged 723.07 ms (SD, 137.70), and the slowest 75% averaged 1,657.05 ms (SD, 367.19; *t*_(16)_ = −13.79; *d *= −3.27; *p *< 0.001).

**Figure 7.**
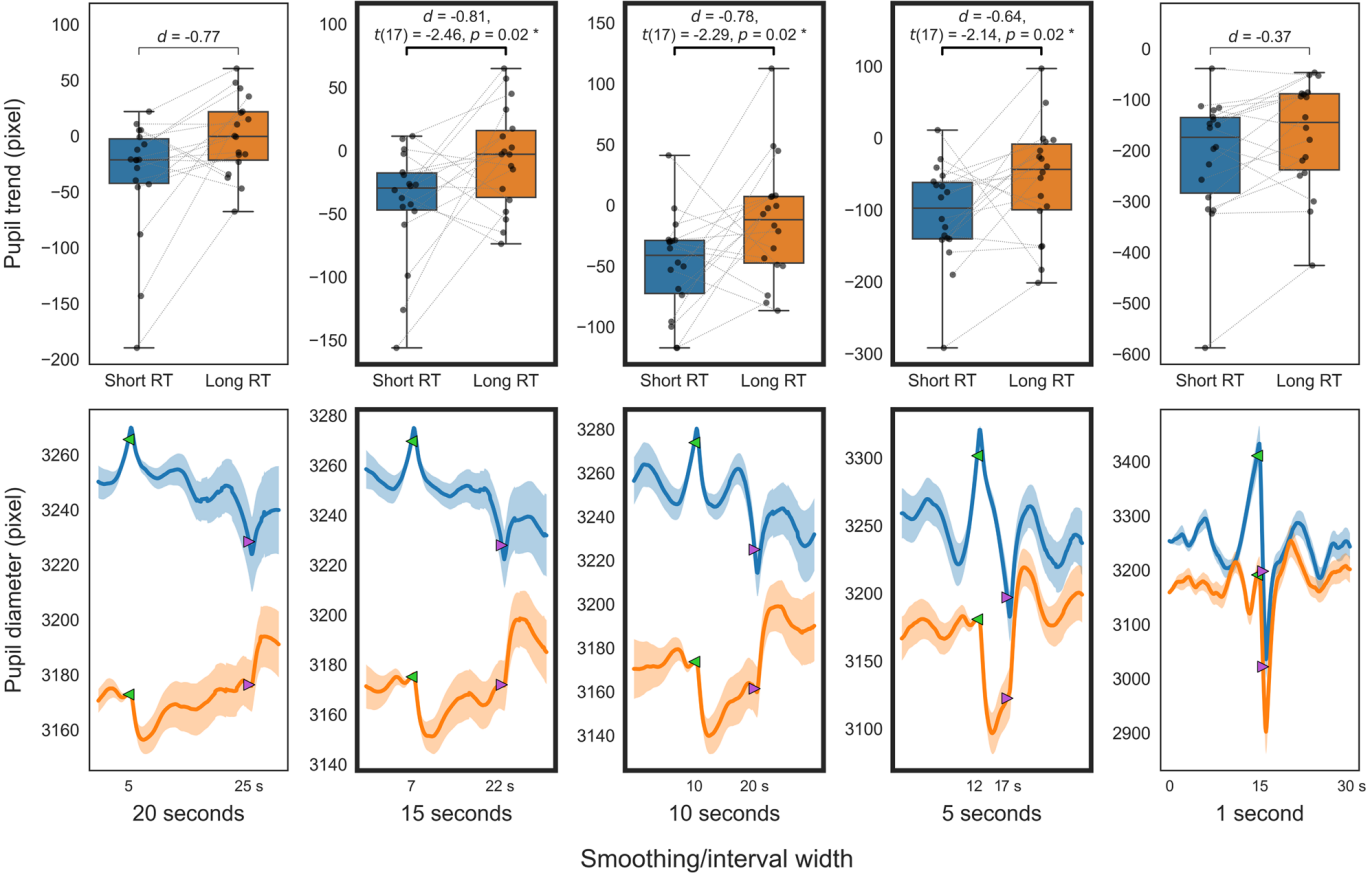
Pupillary comparison in PVT (VG). Smoothed trend (top); smoothed diameter (bottom); between short (blue) and long reaction times (orange). The top panels present box-and-whisker plots of the trend indices. The bottom panels display the smoothed pupil diameters that serve as the source of the trend indices (solid line, mean smoothed pupil diameter for 30 s; translucent area, standard error). For visualization purposes, the smoothed pupil diameter for each participant is adjusted to the average diameter across participants at the start time of trend calculation (green marker).

**Figure 8.**
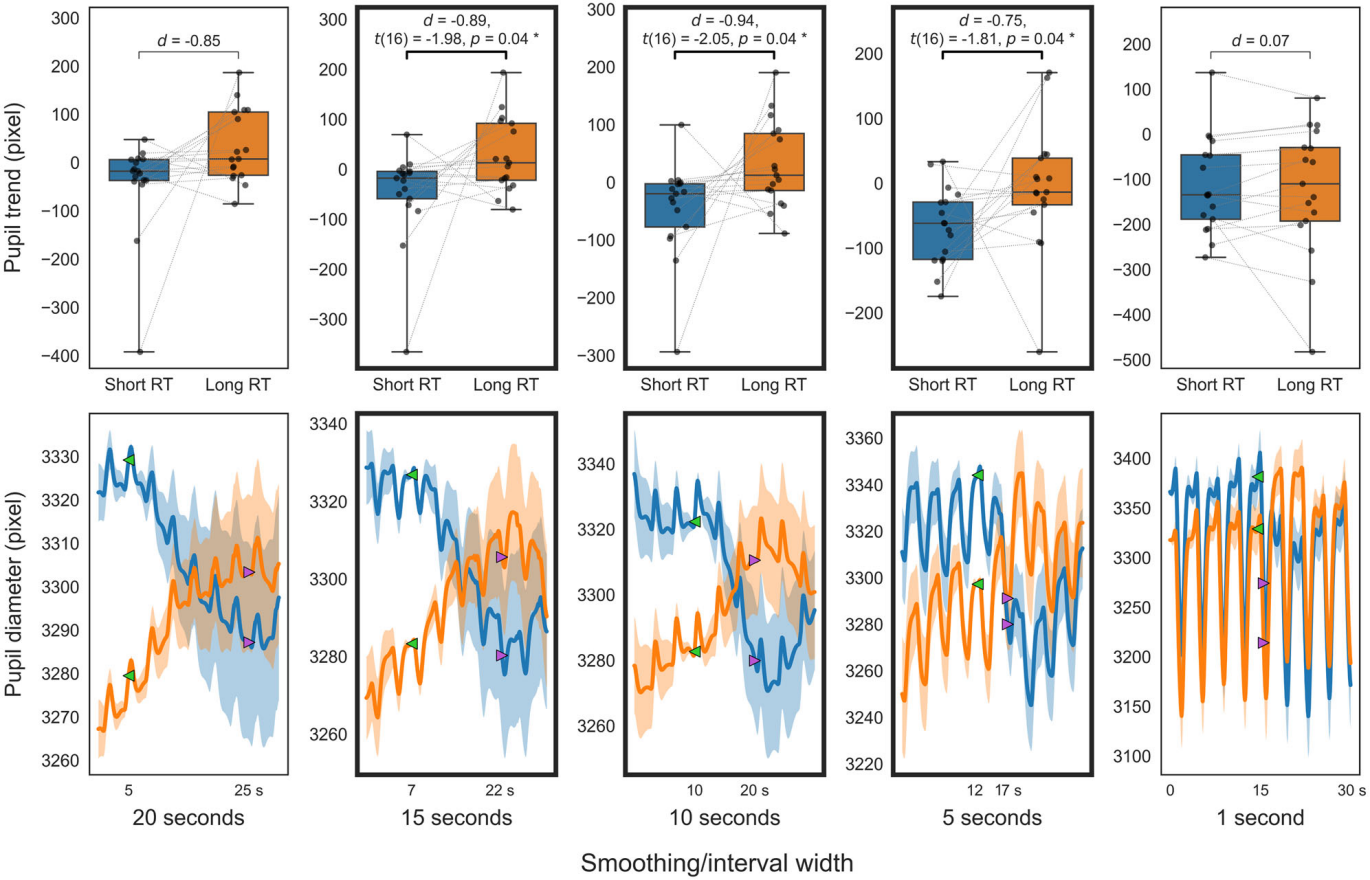
Pupillary comparison in 2BT (WM). Smoothed trend (top); smoothed diameter (bottom); between short (blue) and long RT (orange). The top panels display box-and-whisker plots of the trend indices. The bottom panels show the smoothed pupil diameters that serve as the source for the trend indices (solid line, mean smoothed pupil diameter for 30 s; translucent area, standard error). For visualization purposes, each participant's smoothed pupil diameter is adjusted to the average diameter across participants at the start of the trend calculation (green marker).

**Figure 9.**
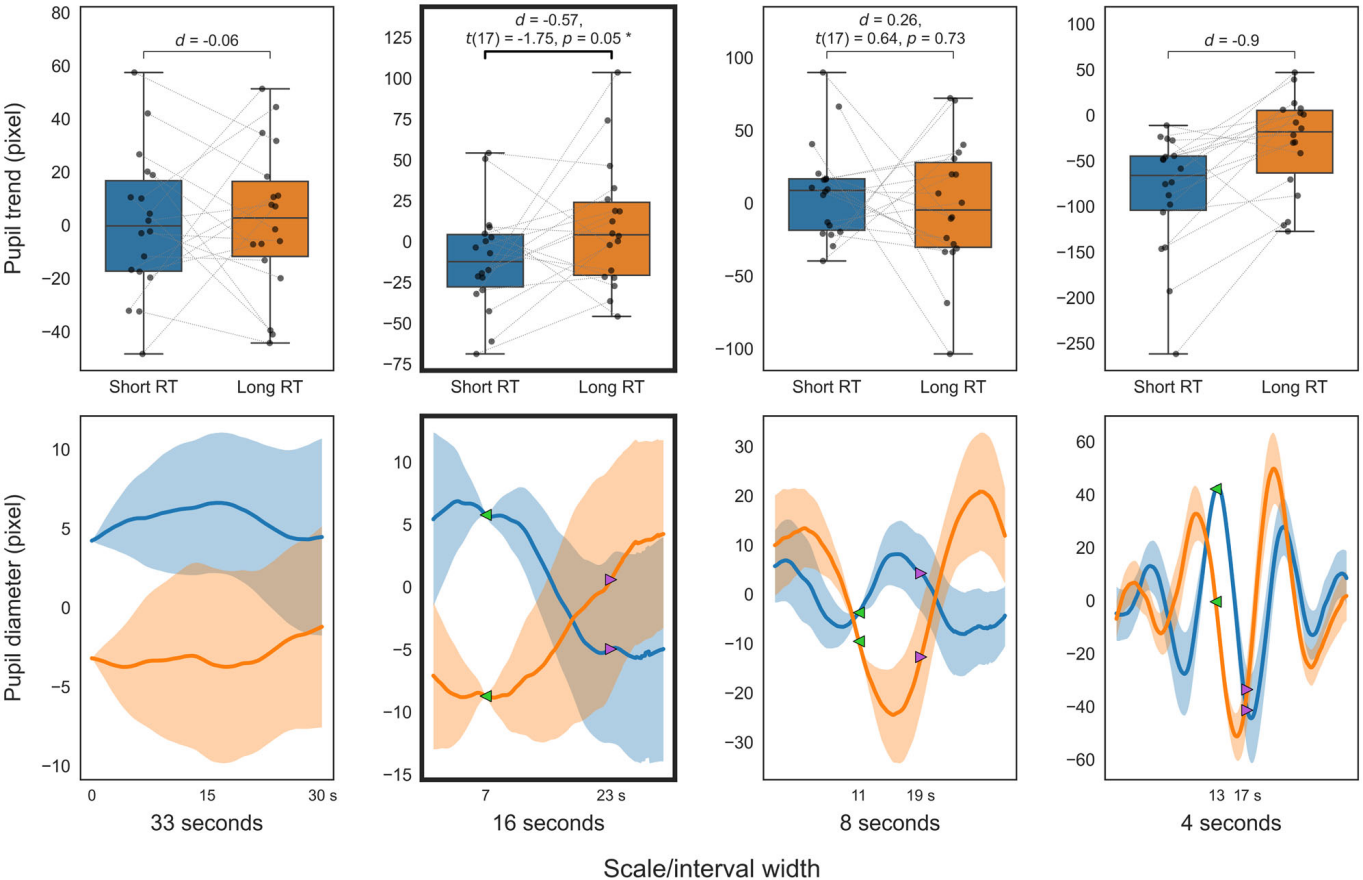
Pupillary comparison in PVT (VG). Decomposed trend (top); decomposed diameter (bottom); between short (blue) and long RT (orange). The top panels present box-and-whisker plots of the trend indices. The bottom panels display the decomposed pupil diameters that serve as the source for the trend indices (solid line, mean decomposed pupil diameter for 30 s; translucent area, standard error). For visualization purposes, the decomposed pupil diameter for each participant is adjusted to the average diameter across participants at the start of the trend calculation (green marker).

**Figure 10.**
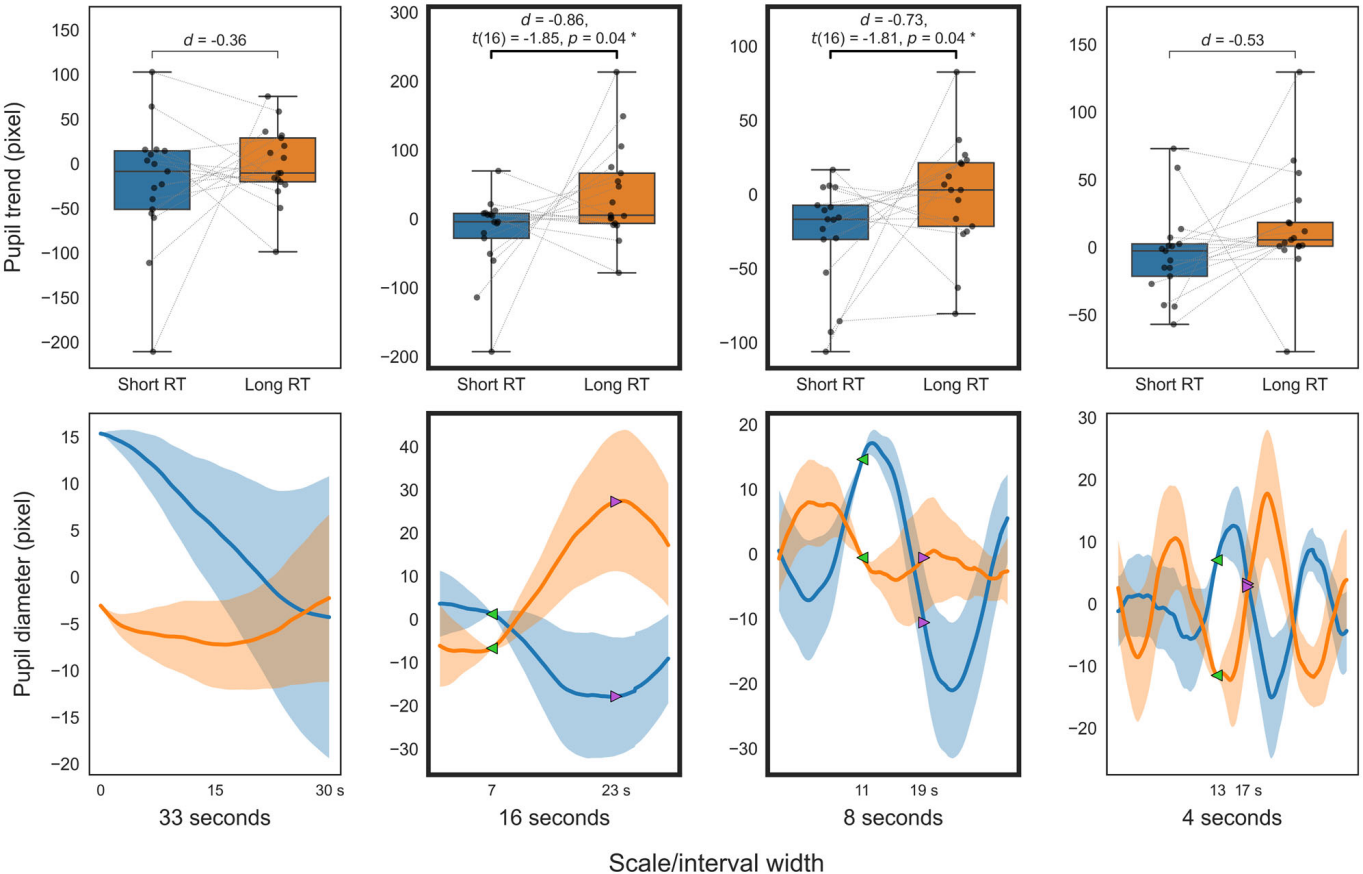
Pupillary comparison in 2BT (WM). Decomposed trend (top); decomposed diameter (bottom); between short (blue) and long RT (orange). The top panels display box-and-whisker plots of the trend indices. The bottom panels show the decomposed pupil diameters that serve as the source of the trend indices (solid line, mean decomposed pupil diameter for 30 s; translucent area, standard error). For visualization purposes, each participant's decomposed pupil diameter is adjusted to the average diameter across participants at the start of the trend calculation (green marker).

The initial analysis of smoothed trends across different temporal scales for PVT and 2BT, as illustrated in Figures 7 and 8, revealed that trends over 5 s windows in short RT trials were significantly lower than in long RT trials (*p *< 0.05; FDR corrected), with a slightly larger effect size observed for the 15–10 s window compared with adjacent windows. Further analysis involved calculating independent pupil trends over these timescales using multiresolution decomposition. The analysis of decomposed trends across different temporal scales for PVT and 2BT, as shown in Figures 9 and 10, respectively, found that trends from 16 (and 8) second scales in short RT trials were significantly lower than in long RT trials (*p *< 0.05; uncorrected).

#### ANT

Finally, we investigated whether the smoothed or decomposed trend index reflected the need for sustained response preparation in the face of temporally unpredictable targets, consistent with the concept of tonic nonoptimality detection and compensation (Sadaghiani and D'Esposito, 2015) rather than phasic detection of deviations (such as stimulus interference in this study).

In the alerting condition, the center-cue scenario, which externally indicated the timing of target presentation, contrasted with the no-cue condition that necessitated the participant's sustained alertness throughout the variable interval, potentially impacting the pupil trend. Mean RT for center-cue trials was 685.10 ms (SD, 144.37), compared with 714.88 ms (SD, 165.39) for no-cue trials (*t*_(16)_ = −3.50; *d *= −0.19; *p *= 0.003).

**Figure 11.**
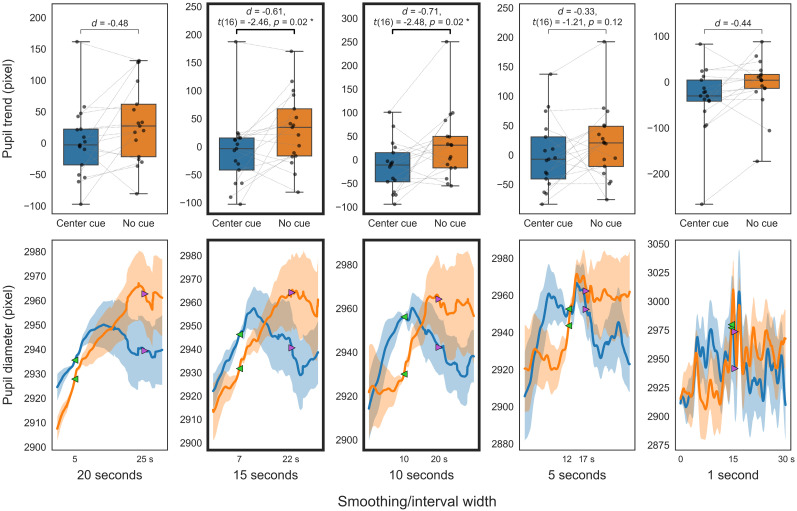
Alerting comparison of the pupil in ANT. Smoothed trends (top); smoothed diameters (bottom). Center cue (blue), no cue (orange). The top panels present box-and-whisker plots of the trend indices. The bottom panels show the smoothed pupil diameters that serve as the source of the trend indices (solid line, mean smoothed pupil diameter for 30 s; translucent area, standard error). For visualization purposes, each participant's smoothed pupil diameter is adjusted to the average diameter across participants at the start of the trend calculation (green marker).

**Figure 12.**
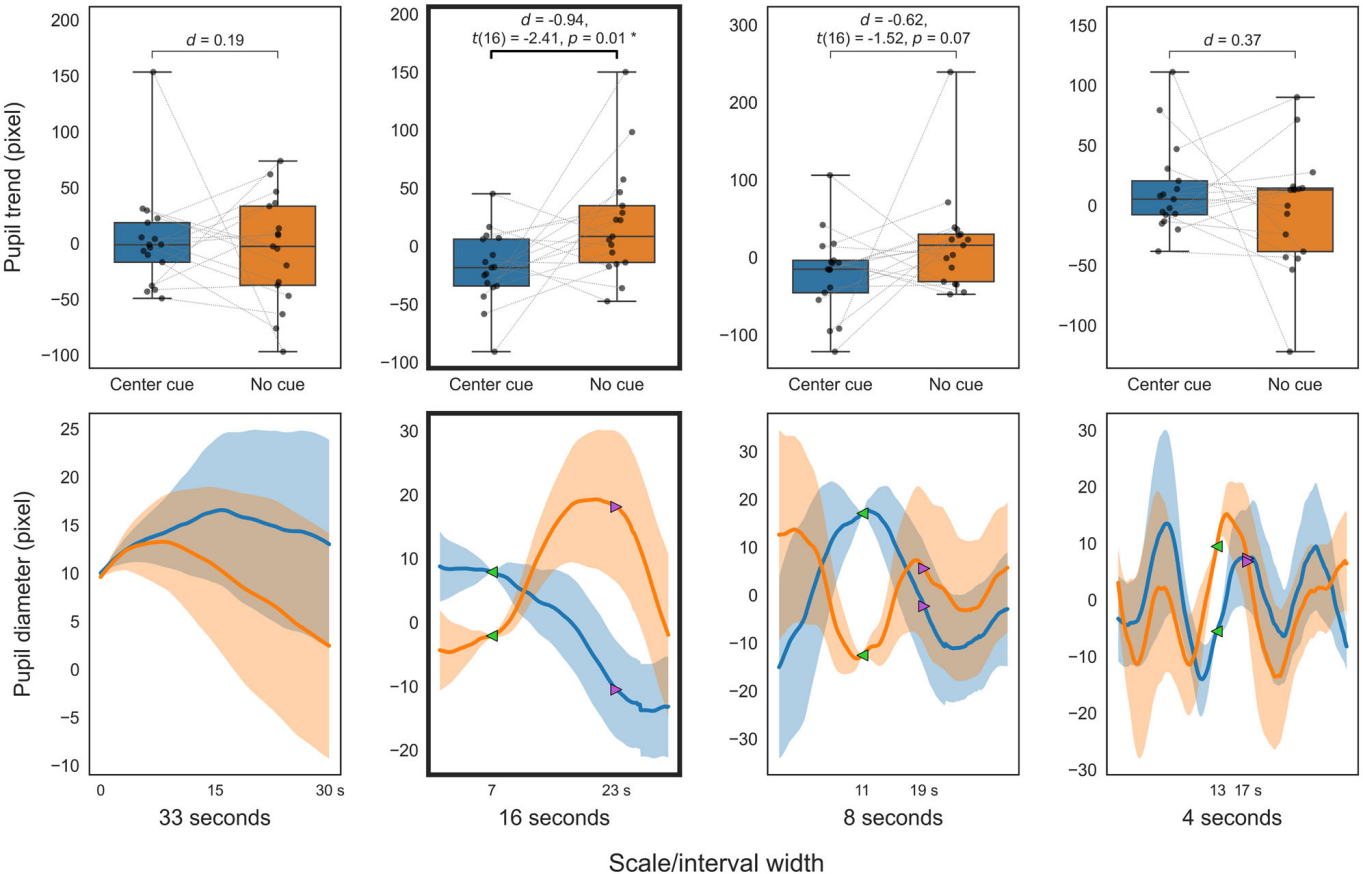
Alerting pupil comparison in ANT. Decomposed trends (top); decomposed diameters (bottom). Center cue (blue), no cue (orange). The top panels present box-and-whisker plots of the trend indices. The bottom panels show the decomposed pupil diameters that serve as the source of the trend indices (solid line, mean decomposed pupil diameter for 30 s; translucent area, standard error). For visualization purposes, each participant's decomposed pupil diameter is adjusted to the average diameter across participants at the start of the trend calculation (green marker).

Analysis of ANT trends using different smoothing windows for the alerting condition (Fig. 11) confirmed our expectations. Significantly lower pupil trends were observed in center-cue trials compared with no-cue trials in the 15 to 10 s windows (*p *< 0.05; FDR corrected). The 10 s trend difference was ∼0.070 mm (51.13 pixels). The conservative trend for a 10 s window also showed a significant difference (*t*_(16)_ = −2.64; *d *= −0.77; *p *= 0.009). In the multiresolution decomposition analysis, trends at different scales in the ANT alerting comparisons (Fig. 12) showed that the trend at the 16 s scale in center-cue trials was significantly lower than in no-cue trials (*p *< 0.05; uncorrected).

**Figure 13.**
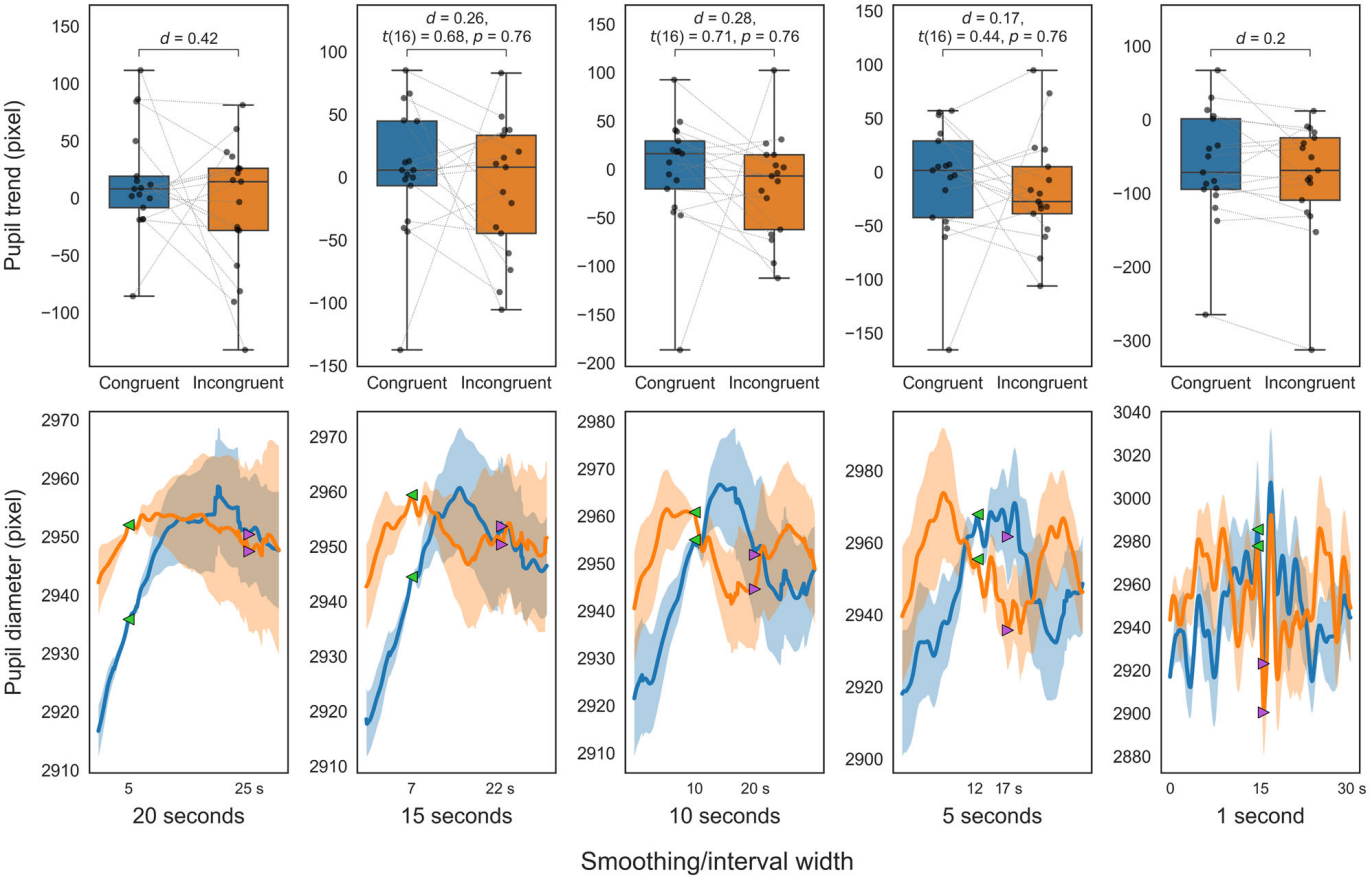
Executive control comparison of pupil trends in ANT. Smoothed trends (top); smoothed diameters (bottom). Congruent target (blue), incongruent target (orange). The top panels present box-and-whisker plots of the trend indices. The bottom panels show the smoothed pupil diameters that serve as the source of the trend indices (solid line, mean smoothed pupil diameter for 30 s; translucent area, standard error). For visualization purposes, each participant's smoothed pupil diameter is adjusted to the average diameter across participants at the start of the trend calculation (green marker).

Conversely, the congruent target condition, lacking interference, differed from the incongruent target condition that entailed interference, a distinction we anticipated would not influence the pupil trend (i.e., executive control comparison). The mean RT for congruent targets was 667.12 ms (SD, 168.45), against 729.26 ms (SD, 160.34) for incongruent targets (*t*_(16)_ = −5.98; *d *= −0.37; *p *< 0.001). The executive control comparison trends (Fig. 13) revealed no significant differences.

## Discussion

The pupil trends identified at timescales >10 s, indicative of suboptimal tonic alertness maintenance, showed a positive correlation with trial-by-trial RTs (i.e., a negative correlation with performance) in the PVT and 2BT. In the ANT, this trend differentiated conditions with and without a timing cue, distinguishing between efficient (optimal) and inefficient (suboptimal) tonic alertness maintenance. Furthermore, the presence or absence of target interference did not influence the trend, indicating that it is unrelated to the phasic alertness response. Our findings support that pupil-linked variations in suboptimal tonic alertness maintenance over a 10 s timescale relate to performance decrements in VG and WM tasks.

Earlier research has thoroughly examined fast pupil changes over a trial, i.e., the serial flow of the start of a trial, the latency or retention period, the target presentation, and a response (Table 2). Studies have shown that “pupil dilations” (i.e., high temporal resolution changes during the latency or retention period) and “phasic responses” time-locked to the target presentation are positively correlated with trial-by-trial performance in VG, WM, and other tasks (Unsworth et al., 2020; Hood et al., 2022). However, this research did not demonstrate the slow pupil changes that might negatively correlate with performance. Previous within-individual correlational analyses using “pretrial (baseline) pupil size” (i.e., the absolute magnitude at the start of a trial) highlighted a gap in evidence for tonic pupil signals linearly related to trial-by-trial VG performance (cf. Introduction; Martin et al., 2022). Most studies relied on pupil indices derived from high temporal resolutions, potentially capturing a wide range of internal states, including tonic alertness maintenance across different timescales, phasic changes, and various emotional factors (Bradley et al., 2008; Unsworth et al., 2018; Unsworth and Robison, 2018; Robison and Unsworth, 2019).

**Table 2.** Summary of tonic and phasic pupil indices in VG and WM tasks

	Long-term (tonic, beyond a trial)	Intermediate (within a trial)	Short-term (phasic, instantaneous)
Pupil size	Pretrial (baseline) size at the start of a trial: Larger variations negatively correlated with performance	(Not applicable)	(Not applicable)
Pupil changes	Pupil trends across several trials: negatively correlated with performance	Pupil dilations between the start of a trial and the target presentation: positively correlated with performance	Phasic pupil responses around the target presentation: positively correlated with performance

On the contrary, our investigation employed pupil trends at lower temporal resolutions, likely isolating signals of suboptimal tonic alertness maintenance over timescales >10 s. This approach allowed the pupil trend to emerge as a distinct biomarker of suboptimal tonic alertness maintenance, especially in cases where alertness regulation fails to return to an optimal level despite continuous effort for >10 s, as seen in VG and WM tasks. Our results align with those of Van Den Brink et al. (2016), which indicated that increased pupil changes over 30 s correlated with reduced VG performance. The effectiveness of slow trend calculations may also shed light on the neural basis of resting-state pupil fluctuations below 0.1 Hz, which are associated with increased fatigue or drowsiness (Wilhelm et al., 1998). Furthermore, our novel index is robust against rapid luminance fluctuations, making it suitable for assessing concentration maintenance across various real-world tasks under different lighting conditions.

### Complementary pupillometric approaches for assessing tonic and phasic alertness

Although traditional pupil indices (e.g., pretrial pupil size, pupil dilations, and phasic pupil responses) capture a broad range of internal states, they may not effectively differentiate the specific temporal aspects of tonic alertness maintenance. These conventional indices could support integrative theories, such as the LC–NE account of attention control and WM (Unsworth and Robison, 2017), by elucidating the function of between-individual differences. This framework posits that individuals exhibiting significant within-individual variations in tonic alertness (pretrial pupil size) tend to have lower phasic (pupil) response and poorer performance on average in between-individual analyses. The conventional index's ability to capture broad performance variations across participants sufficed for between-individual differences analyses (Unsworth and Miller, 2021). However, accurately capturing the nuanced details of within-individual tonic alertness fluctuations for different temporal resolutions may necessitate a more refined approach.

Introducing the pupil trend metric offers the advantage of capturing the detailed characteristics of slow changes in internal states, specifically different timescales of tonic alertness fluctuations. In our approach, differences in the low-resolution pupil index, without corresponding high-resolution differences, suggest slow tonic alertness fluctuations after excluding fast pupil changes caused by various factors. This study demonstrates how isolating suboptimal tonic alertness maintenance over 10 s helps elucidate trial-by-trial alertness regulation within individual performance across key VG and WM tasks.

Traditionally, tonic alertness has been assumed to occur prior to trial initiation and is inferred from pretrial pupil diameter measurements. This approach, however, may limit tonic alertness detection to the period before the latency or retention phases, potentially overlooking its active presence closer to the target presentation. The findings from this study suggest that variations in tonic alertness maintenance are not merely passive phenomena occurring well before the target onset but, instead, actively manifest around the time of target processing.

### Possible mechanisms of internal alertness regulation

Given the roles of the AI/FO, LC–NE, and thalamus, the observed pupillometric patterns likely reflect the regulation of intrinsic alertness to counteract decreased tonic alertness caused by internal (PVT and 2BT) or external (ANT) factors. Overall (tonic) alertness affecting performance can be influenced by three factors: internal voluntary (i.e., intrinsic alertness), internal involuntary (e.g., fatigue), and external (e.g., uncertainty in target presentation) factors (Sturm and Willmes, 2001). These neural structures may regulate intrinsic alertness, helping to transition from nonoptimal to optimal states for task performance (Corbetta and Shulman, 2002; Menon and Uddin, 2010; Sterzer and Kleinschmidt, 2010; Tian et al., 2014; Sadaghiani and D'Esposito, 2015).

In this transition, the AI/FO may detect internal involuntary nonoptimality, such as cognitive fatigue (CF; Anderson et al., 2019) or default mode network (DMN; Sridharan et al., 2008) activation (Ullsperger et al., 2010). It may also detect external nonoptimality, such as the necessity for, and the detrimental impact on, maintaining response readiness during unpredictable intervals (Sadaghiani and D'Esposito, 2015). Detection signals from the AI/FO, monitored by higher cognitive functions, may gradually enhance intrinsic alertness to mitigate these adverse effects through the regulatory influence of the LC–NE and thalamus (Langner and Eickhoff, 2013; Sadaghiani and D'Esposito, 2015).

When a participant's overall alertness is significantly reduced due to internal or external factors, restoring intrinsic alertness to an optimal state may take 10 s or more (i.e., suboptimal alertness maintenance). This finding could explain the 10 s pupil increase associated with below-average performance. The tonic signals lasting over 10 s, identified in this study, may reflect the regulation of intrinsic alertness in response to nonoptimal factors during VG and WM tasks. Variations in CF and DMN activity, potentially mirrored in AI/FO activity (Sterzer and Kleinschmidt, 2010; Langner and Eickhoff, 2013; Anderson et al., 2019), could influence compensatory pupil trends that correlate with RTs in PVT (VG) and 2BT (WM) tasks. In complex tasks like VG and WM, intrinsic alertness regulation may not fully compensate for nonoptimality detected by the AI/FO due to involuntary or external factors, leading to a negative relationship between pupil trends and task performance.

Furthermore, the AI/FO's role in distinguishing the need for maintaining response readiness in the no-cue condition but not in the center-cue condition of the ANT may explain the observed differences in pupil trends between these conditions. In the ANT, when a no-cue trial appears amid repeated trials indicating target presentation timing, alertness may drop significantly below the (increased) level required for task performance. Since this alertness deficit is not instantaneous, as in interference resolution, but persistent (tonic), intrinsic alertness regulation lasting >10 s may be activated after the perception of cue absence (cf. Sadaghiani and D'Esposito, 2015).

This tonic alertness regulation, occurring over 10 s (i.e., across several trials), may establish the prerequisite baseline for alertness regulation within shorter periods (i.e., a single trial; Table 2). In the nonoptimal range, the process of recruiting alertness may typically take 10–15 s, after which intermediate and phasic regulation can be effective. With the alertness around the optimal range, intermediate regulation may help transition overall alertness from slightly suboptimal to optimal, focusing on target presentation timing. These different roles of tonic and intermediate alertness regulation may explain the observed correlations: baseline adjustment (pupil trend) negatively correlates with performance, while intermediate adjustment (pupil dilation) positively correlates with performance. These discussions may also contribute to a better understanding of attention and WM, accounting for how significant within-individual tonic alertness variations lead to poorer overall performance per individual (Unsworth and Robison, 2017).

Moreover, after such regulation, the AI/FO-related networks are known to instantly detect externally evoked interference (i.e., phasic response), such as a more or less unexpected (salient) target appearance during a continuous preparatory state (Corbetta and Shulman, 2002), task-relevant targets emerging amid other internal activities like DMN activation (Sridharan et al., 2008; Menon and Uddin, 2010), or targets surrounded by distracting stimuli (Eckert et al., 2009). The pupil trend differs from pupil changes reflecting phasic responses due to its temporal resolution and because it showed no effect in the executive control comparison in the ANT. Since this remains speculative, future studies are needed to fully capture the temporal dynamics of alertness regulation by analyzing tonic (pupil trend), intermediate (pupil dilation), and phasic (phasic pupil response) changes, thus covering the spectrum of internal nonoptimality, suboptimality, and optimality.

### Limitations and future directions

This study is primarily an exploratory study with a small sample size, necessitating confirmatory analyses to validate the current hypotheses using a larger sample. We speculate that the primary effects (e.g., pupil trend differences over >10 s) should be replicable in future studies, as they generally showed sufficient statistical power in the current sample (*d *< −0.63; *N* = 17; 80% power, one-tailed). However, we could not investigate the exact scale at which the maximum effect occurs or the lower boundary at which the effect remains due to the limited statistical power (sample size) available for this exploratory study. Indeed, the nonsignificant effects in these ranges (−0.63 < *d *< 0) may become significant with a larger sample size.

Additionally, the various analyses conducted to ensure consistency and robustness (Steegen et al., 2016) may raise concerns about the familywise error rate due to multiple comparisons. This study corrected multiple comparisons for tests using strongly correlated variables (see FDR corrections for smoothed trends). From this perspective too, future studies are needed to confirm the current exploratory findings.

While this study is currently exploratory, it highlights the need for further research to elucidate these mechanisms comprehensively. Integrating pupil trend analysis with brain imaging techniques could unravel the central and autonomic nervous systems’ roles in modulating tonic and internal states of concentration during task execution. Moreover, the resilience of pupil trends to rapid luminance fluctuations might shed light on concentration dynamics in more naturalistic tasks, where stimulus luminance variations are less predictable.

## Conclusion

This study tested the hypothesis that variations in tonic alertness maintenance underlie trial-by-trial performance in VG and WM tasks. We demonstrated that pupil trends lasting >10 s, indicative of suboptimal tonic alertness maintenance, are negatively correlated with performance on a trial-by-trial basis in both task types. Additionally, we provided convergent evidence that these pupil trends reflect suboptimal alertness maintenance persistently induced rather than phasic responses triggered by explicit interference events. These exploratory findings suggest that intrinsic alertness over a 10 s timescale is a critical factor influencing performance variations in VG and WM tasks.

## Synthesis

Reviewing Editor: Ifat Levy, Yale School of Medicine Decisions are customarily a result of the Reviewing Editor and the peer reviewers coming together and discussing their recommendations until a consensus is reached. When revisions are invited, a fact-based synthesis statement explaining their decision and outlining what is needed to prepare a revision will be listed below. The following reviewer(s) agreed to reveal their identity: Nash Unsworth.

The authors have adequately addressed all comments.

## Author Response

Dear Dr. Ifat Levy and the Reviewers, We sincerely thank Dr. Levy and the Reviewers for their thoughtful review of our paper entitled "Pupil Trend Reflects Sub-Optimal Alertness Maintenance Over 10 Seconds in Vigilance and Working Memory Performance: An Exploratory Study." We appreciate the opportunity to resubmit the manuscript and are grateful for the significant impact of your insightful comments on improving the paper. We also thank you for extending the deadline for resubmitting the manuscript.

Below, we address the Editor's and Reviewers' comments. We have attached the revised manuscript at the end of this response sheet. All line numbers referenced in this response correspond to those in this version. Any deleted or inserted text is highlighted. (We have also attached a manuscript that tracks only the insertions as a separate file, as required by the editorial office.) Response to the Code Accessibility Comments ---- Please include a statement in the Materials and Methods section, under the heading "Code Accessibility", indicating whether and how the code can be accessed, including any accession numbers or restrictions, as well as the type of computer and operating system used to run the code.

---- We thank you for your comment. We have added the "Code Accessibility" section (lines 292-297) indicating that "the data analysis code/software is freely available online at [URL redacted for double-blind review]. Researchers can use the code under a license set by [author's organization], which allows them to use it for scientific evaluation." The editorial office can check the clean copy version (not anonymized) for the actual URL, outside the double-blind review. We have also noted the type of computer and operating system on which the code was run.

Response to Synthesis Statement ---- 1) The authors should expand on what the pupil trend measure is, how it is computed, and how it relates to tonic alertness ...

---- We appreciate your feedback and have expanded the description of the trend measure in the revised manuscript (lines 258-265 and 288-291), adding new Figures 4 and 5. We have further clarified how this measure relates to tonic alertness (i.e., task performance), as shown in the new bottom panels of Figures 7-13.

---- 1-A) ... , for example: What is a negative value for the trend, and what is a positive value? Is it a negative/positive change over time? Is it a mean over the window? a linear slope? A non-linear trend? ...

---- We explained that the pupil trend represents the increase or decrease from the smoothed/decomposed diameter at an earlier time point to a later one as follows Regarding the smoothed trend (see also Figure 4): "For example, with a 10-second window, the change in pupil diameter over this period was quantified by measuring the increase or decrease. The change was calculated by subtracting the smoothed pupil diameter at the beginning of the 10-second window from that at the end. The value at the start represents the smoothed pupil diameter 5 seconds before the target presentation (i.e., the average of the pre-smoothed pupil diameter from -10 to 0 seconds), and the value at the end represents the smoothed pupil diameter 5 seconds after the target presentation (i.e., the average of the pre-smoothed pupil diameter from 0 to 10 seconds)" (lines 258-265). "This is calculated by subtracting the diameter indicated by the green marker from that indicated by the purple marker" in the right middle panel of Figure 4 (lines 878-879).

Regarding the decomposed trend (also see Figure 5): "For instance, at an 8-second resolution, the change in pupil diameter over this period was quantified. By examining the time series data at an 8-second temporal resolution, we compared the pupil diameters 4 seconds before and after the target presentation. The trend was calculated by subtracting the earlier value from the later one" (lines 288-291). Again, "the trend calculation subtracts the decomposed diameter indicated by the green marker from that indicated by the purple marker" in the right middle panel of Figure 5 (lines 891-892).

---- 1-B) ... If so, what is that being compared to? ---- "In the data analysis, we first presented the trial-by-trial differences in pupil trends between varying VG and WM performances" (lines 299-300). Specifically, these trends for short RT and long RT trials were summarized separately, and we then compared the difference between these two indices. For example, "an analysis was conducted to determine if average pupil trends varied between these performance levels" (lines 338-339).

---- 1-C) What might help is a visualization of the effect. For example, comparing short and long RTs in the PVT in the 10-second scale, for which there was the biggest effect, could you show the whole waveform? The single value on the y-axis of the figures did not really make much sense to me.

---- We have added the bottom panels of Figures 7-13, which display the whole waveform of the smoothed/decomposed pupil diameter for 30 seconds centered on the moment of the target display in PVT, 2BT, and ANT.

---- 2) Does the pupil trend analysis perform any sort of baseline correction, or standardization? Or does it leave pupil diameter in the arbitrary units in which the EyeLink measures pupil? ---- No correction or standardization was performed; the trend was calculated based on the difference between the raw magnitude values at the two time-points. The units are in pixels, as recorded by EyeLink's camera. We chose not to apply preprocessing because, for future practical applications, we aimed to develop a method that requires minimal preprocessing and parameter setting.

For reference, we have provided a rough estimate of the effect size in the revised manuscript: "Although we did not develop a precise transformation formula, it is worth noting that the 10-second trend differences (59.23 pixels in the PVT and 56.61 pixels in the 2BT) were approximately 0.07-0.08 mm. This estimation is based on the assumption that the mean pupil size at the PVT target presentation of 3,300 pixels corresponds to a generally observed pupil size of 4.5 mm against a dark background in a dimly lit room (Peysakhovich et al., 2015)" (lines 363-368). In the ANT, "The 10-second trend difference was approximately 0.070 mm (51.13 pixels)" (lines 407-408).

---- 3) Why would a larger pupil trend be associated with longer RTs? What exactly does this mean? Why would a larger pupil trend be associated with lowered tonic alertness? Based on some of the studies the authors cite, which look at baseline pupil diameter wouldn't you predict that lowered pupil trend (associated with lowered arousal and perhaps alertness) would be associated with longer RTs? The authors need to explain the seemingly contradicting results, and why they predicted them (Table 1).

---- We appreciate the reviewers' insightful comments and understand the need to clarify the relationship between baseline pupil size and the pupil trend to reduce potential confusion.

The apparent contradiction can be addressed by considering that a larger baseline is more likely to be associated with a smaller trend, rather than a larger one (see Figures 7 and 8). This is because when the baseline pupil size is already large, the trend (i.e., the increase in diameter) is limited by the physiological upper bounds of pupil diameter. Conversely, a large trend is more likely to occur when the baseline is smaller, which aligns with the expectation of longer RTs and lowered arousal (or perhaps alertness).

While this explanation is consistent with intuitive reasoning, our original prediction was rooted in neuroscientific findings (lines 49-50). Based on previous research, we expected a negative correlation between a slow increase in pupil diameter and task performance (lines 58-66). However, existing studies on baseline pupil size present mixed evidence regarding whether increases or decreases in pupil size are more strongly linked to performance (lines 81-86). This inconsistency is likely due to the broad range of timescales considered in these studies, highlighting the need to explore different timescales in more detail (lines 86-88).

To address these issues, we have revised the Introduction to clearly differentiate the pupil trend from baseline pupil size and to emphasize the importance of examining specific timescales (e.g., 10 seconds) to better understand their relationship with task performance (lines 81-88 and 92-94). In addition, we have added Figures 7-8 that suggest an intuitive description based on the physiological limits mentioned above. Additionally, throughout the manuscript, sentences have been revised to better match these explanations (the revised title, lines 4-7, 20-23, and elsewhere).

---- 4) How exactly were the pupil-trend values used to examine differences in task performance? Also, please provide more details on why were RTs split at the mean. Could this have impacted the results? Is it possible that looking at more extreme RTs (fastest 25% vs. slowest 25%) would have given different results for the different time windows? ---- Thank you for the opportunity to clarify the procedure and for your suggestion of ways that can strengthen our analysis. Following the reviewers' comments, we conducted additional comparisons using different criteria and found that the results remained consistent.

In this study, we divided the trials into two groups within individuals based on response (e.g., RT) and compared the trend indices between them (see also point (1-B)). We made this split because we were interested in the within-individual differences between good and poor performance states in VG and WM tasks. However, the criterion for the split was not arbitrary.

To eliminate any potential bias, we have added a new "primary analysis" (lines 369-390) in the revised manuscript as suggested by the reviewers while retaining the original mean split analysis as a "preliminary analysis" (lines 334-368; see also 299-302). The primary analysis compared the trends between the fastest 25% of trials and the slowest 75% of trials to ensure a more rigorous comparison. The results of this primary analysis (Figures 7-10) showed the same effects as the original/preliminary analysis (Figure 6).

Note that the slight changes in the statistics in the original/preliminary analysis (e.g., lines 344-350) are due to a minor correction in the analysis code, which does not affect the main results (see "Changes not included in the response to the reviewers" section at the end of this response sheet).

---- 5) Specifically for the ANT, it is not clear how the pupil trend is differentiating between trial types. It seems that the 10-second window, for example, is centered on stimulus presentation. Thus, it includes 5 seconds of pre-stimulus activity and 5seconds of post-stimulus activity. There is no way for a person (or their cognitive system) to differentiate between a congruent or incongruent upcoming trial - is the argument that the 5-seconds of post-stimulus activity following different trial types drives the difference in the 10-second pupil trend? ---- We agree that the post-stimulus (post-target) pupil activity largely drives the 10second trend difference in the ANT (see the added bottom panel of Figure 11). However, we would like to clarify that the interval during which the post-stimulus pupil diameter differs is not 5 seconds but 10 seconds (see the right middle panel of Figure 4).

---- 5-A) If so, how is that not clouded by different phasic responses to different trials? To ensure that our results are not confounded by phasic responses, we have added a conservative analysis to show the trend differences while excluding the pupil changes around the target presentation (see lines 341-342). Since the 10-second smoothed trend subtracts the average pre-smoothed pupil diameter from −10 to 0 seconds from the average pre-smoothed pupil diameter from 0 to 10 seconds (see the right middle panel of Figure 4), we alternatively calculated the "conservative trend," which subtracts the mean raw pupil diameter between −12 to −2 seconds from the mean raw pupil diameter from 2 to 12 seconds (see lines 266-273). We used this measure to carefully exclude the effects of phasic activity that could occur within a 4-second range around the target presentation (see the effect of slight abrupt changes seen even in smoothed pupil diameter in Figures 7 and 8). As a result, the primary effects of the 10-second trend remained (see lines 359- 360 and 407-409). Therefore, the revised manuscript demonstrates that the 10-second smoothed trend persists, irrespective of phasic responses within 4 seconds.

---- 5-B) Also, for all trial types, are the wide windows, which include post-stimulus activity, not clouded by other processes like error monitoring? ---- Given the small number of explicit errors participants could monitor and the complete exclusion of these errors, error monitoring did not affect the results. In our understanding, error monitoring is the process of recognizing whether one's response is incorrect after each trial. However, this study's explicit errors (i.e., incorrect responses) were very rare (9/2,088 in PVT, 239/2,435 in 2BT, and 58/1,632 in ANT). After excluding all these error responses in the revised manuscript (e.g., lines 309 in PVT, 322 in 2BT, and 329-331 in ANT), only trials remained where participants could not objectively identify their responses as errors. Except for the PVT, participants were not even given feedback on the length of their RTs. Based on these points, the revised manuscript suggests that error monitoring did not affect the results (the slight changes in the number of trials are due to a minor correction, see "Changes not included in the response to the reviewers" section).

Nevertheless, it is possible that participants were aware that their internal state was somehow non-optimal for performing the task and may have adjusted their alertness voluntarily during the post-target period. We addressed this point in response to point (8). ---- 6) The sample size is very small, even for the within-individual effects.

---- We acknowledge the reviewers' concern about the small sample size. Given the scope of this study, we designed it as an exploratory analysis, rather than a confirmatory one. To reflect this and to be statistically fair, we have revised the manuscript to clearly state that this is an exploratory study (e.g., the revised title), instead of the post-hoc increment of participant number or replication in a larger sample.

---- 6-A) At this sample size, the authors only have sufficient power (80% power, alpha = .05, two-tailed) to detect effect sizes of .70. Further, some of the non-significant effect sizes are quite large (d = .30-.50).

---- We agree that the sample size is only sufficient to detect large effect sizes for around 10-second trends (see also point (6-D) for power analysis results), meaning that more minor effects may become significant in future studies. We explicitly mentioned this limitation in the "Limitations and Future Directions" section (lines 622-626). We also revised the manuscript more rigorously regarding the interpretation of these nonsignificant effects (i.e., the extended time range over which the effect potentially occurs, lines 12-16, 20-23, 46-48, 352-353, 436-439, and elsewhere).

---- 6-B) Moreover, the sheer number of ways in which the data are analyzed and sliced up raises the issue of multiple comparisons and familywise error rate; it seems that there was no correction for multiple comparisons.

---- We recognize the potential risk of increased familywise error rate due to the number of analyses performed. We agree that the analysis description in the original manuscript could be interpreted as not having any multiple comparison corrections. We have clarified whether or not the correction was applied for all statistical tests (lines 356, 358, 363, 384, 390, and elsewhere). We decided to perform corrections between tests of smoothed trends using strongly correlated pupil parameters (e.g., FDR correction between the three tests for similar pupil waveforms in Figure 7 or 8). We also mentioned this as the limitation of this study (lines 627-631).

6-C) Finally, there were no directional hypotheses for any of the measures, which means an effect in either direction was interpreted as being consistent with "variations in tonic alertness" (P. 21).

---- We appreciate the reviewers' comments and agree that the hypothesis could be interpreted as two-directional in the original manuscript. Throughout the manuscript, we have highlighted that this is one-directional (line 356; see also Table 1, lines 60-66, 74- 76, and 92-94).

---- 6-D) The authors are strongly encouraged to collect more data, and provide power analysis to justify the sample size. Without additional data, the authors should explicitly mention that the analysis is exploratory, and acknowledge the limitation of the small sample size, and the need for confirmation in a larger sample.

---- We thank the constructive suggestions of the reviewers. To be statistically fair, we selected the option to explicitly mention that this is an exploratory study (the revised title, lines 9-12, 92-94, 647-649), add the limitation section, and state the need for future confirmation (lines 618-619, 632). We also conducted a post-hoc power analysis, confirming that the primary effects in this study exceeded the threshold for sufficient statistical power (lines 619-622).

---- 7) With such a small sample, the correlation analyses across individuals does not seem informative. Additional data is needed in order for the authors to include these analyses; otherwise they should be dropped.

---- We appreciate the reviewers' suggestion regarding the limitations of the sample size. We acknowledge that the sample size is insufficient to provide robust or meaningful insights from correlation analysis across individuals. Therefore, as suggested, we have removed the correlation analysis across individuals from the manuscript (see lines 187- 190 and 421-434).

8) Could you provide further explanation for what you mean by tonic alertness? On p. 3 it is stated that this is the "need to internally maintain response preparation." While some prior work has also used this definition, other research would probably call this "intrinsic alertness" (Sturm &Willmes, 2001). Tonic alertness might include this internal and voluntary maintenance of readiness, but it might also include other factors, such as lowered arousal due to fatigue which would impact the overall readiness to respond, but would not necessarily be influenced by controlled aspects of alertness. Thus, some additional clarification of what is meant by tonic alertness would be helpful.

---- We thank the reviewers for providing opportunities to clarify the concept of "tonic alertness." To address potential confusion and align with a broader understanding of the term, we first renamed the original term "tonic alertness" as "tonic alertness maintenance" (intrinsic alertness; lines 52-58 in p.3), which is separated from the persistent adverse factors that increase the need for maintenance (lines 58-60; see also 9-12, 16-20, 436-441, and elsewhere). In the Discussion, we then categorized the factors affecting (overall) tonic alertness into "three factors: internal voluntary (i.e., intrinsic alertness), internal involuntary (e.g., fatigue), and external (e.g., uncertainty in target presentation) factors (Sturm &Willmes, 2001)" (lines 525-528). We also explained that the 10-second pupil increase "likely reflect the regulation of intrinsic alertness to counteract decreased tonic alertness caused by internal (PVT and 2BT) or external (ANT) factors" (lines 523-525). The involuntary effects may include cognitive fatigue (PVT/2BT), default mode network activation (PVT/2BT), or the task condition requiring sustained response readiness during unpredictable intervals (ANT, lines 533-544).

Finally, we proposed that "When a participant's overall alertness is significantly reduced due to internal or external factors, restoring intrinsic alertness to an optimal state may take 10 seconds or more (i.e., sub-optimal alertness maintenance). This finding could explain the 10-second pupil increase associated with below-average performance" (lines 549-552). "In the ANT, when a no-cue trial appears amid repeated trials indicating target presentation timing, alertness may drop significantly below the (increased) level required for task performance. Since this alertness deficit is not instantaneous, as in interference resolution, but persistent (tonic), intrinsic alertness regulation lasting more than 10 seconds may be activated after the perception of cue absence (cf. Sadaghiani &D'Esposito, 2015)." (lines 566-571).

9) The novelty of the study may be overstated given that previous studies have looked at wide ranges of time preceding stimulus onsets. For example, Hood et al. (2022) examined 5-second wide epochs preceding stimulus onsets in the antisaccade task and showed both within- and between-person variation, and Unsworth et al. (2020) showed similar findings in the PVT. These studies both examined changes over relatively wide windows. Please clarify whether and how the "pupil trend" metric substantially differs from, and is more informative than, the ways in which prestimulus pupil changes were analyzed in those and other studies examining intrinsic/tonic alertness.

---- We appreciate the reviewers' feedback regarding the potential overlap with previous studies. To avoid overstating our study's novelty, we have clarified how our approach differs from those of Hood et al. (2022) and Unsworth et al. (2020).

As shown in the newly added Table 2, the novelty of our study lies in identifying a pupil change negatively correlated with performance at a specific large temporal resolution (see the "Long-term (tonic)" column). While pretrial (baseline) pupil size shows both negatively and positively correlated components over a wide range of timescales (i.e., larger variations negatively correlated with performance), previous studies, such as Hood et al. (2022) and Unsworth et al. (2020), have only found that high temporal resolution pupil dilations within a trial are positively correlated with improved performance (see the "Intermediate" column). In contrast, our study found that low temporal resolution pupil changes across several trials, i.e., occurring once or less every 10 seconds, are negatively correlated with performance (see the "Pupil trends across..." box). As shown in Figure 4, pupil diameter at high temporal resolution increases from -2 to 0 seconds (left panel), while it decreases at low temporal resolution (right panels), highlighting how these two measures can yield different results. These explanations, along with suggested references, have been incorporated into the revised manuscript (lines 446-452 and 463-465).

We also discussed the function of alertness regulation reflected in low temporal resolution pupil changes (pupil trends in this study) compared to high temporal resolution changes (pupil indices in previous studies). The pupil trend appears to be "a distinct biomarker of sub-optimal tonic alertness maintenance, especially in cases where alertness regulation fails to return to an optimal level despite continuous effort for more than 10 seconds" (lines 465-468). "This tonic alertness regulation, occurring over 10 seconds (i.e., across several trials), may establish the prerequisite baseline for alertness regulation within shorter periods (i.e., a single trial; see Table 2). In the non-optimal range, the process of recruiting alertness may typically take 10-15 seconds, after which intermediate and phasic regulation can be effective. With the alertness around the optimal range, intermediate regulation may help transition overall alertness from slightly sub-optimal to optimal, focusing on target presentation timing. These different roles of tonic and intermediate alertness regulation may explain the observed correlations: baseline adjustment (pupil trend) negatively correlates with performance, while phasic-like adjustment (pupil dilation) positively correlates with performance. These discussions may also contribute to a better understanding of attention and working memory, accounting for how significant within-individual tonic alertness variations lead to poorer overall performance per individual (Unsworth &Robison, 2017)." (lines 572-583).

While our study offers a new empirical perspective on the dynamics of tonic alertness, we recognize that these findings are still hypothesis-generating (lines 613-616). We have noted in the manuscript that further research is needed to validate these observations and to expand our understanding of how different temporal resolutions of pupil dynamics relate to cognitive performance (lines 632-633).

Response to Minor Issues ---- - On P. 10 it is stated that participants encountered 160 targets across 158 trials. That must be a typo.

---- Thank you for your suggestion. In the 2-back task, the first two targets did not require responses, resulting in a discrepancy between the number of targets and the number of trials by two. We have added a note in the manuscript to clarify this and prevent misunderstanding (line 213).

---- - The use of parentheses to describe different patterns is confusing. For example, on P. 15: "the pupil trend...showed a positive (negative) correlation with trial-by-trial RTs (performance) in PVT and the 2BT." Another example is on P. 20, "In other words, a higher (lower) task difficulty biases the maximal point toward smaller (larger) values, resulting in a negative (positive) linear relationship between tonic alertness and task performance." It's probably better to break those up into different clauses for clarity.

---- Thank you for your comment. We have revised all sentences using parentheses, and divided them into separate clauses for clarity (lines 437-438) or deleted (lines 592- 594).

Changes not included in the response to the reviewers: • We corrected minor errors in the analysis code, resulting in slight changes to the overall statistical results. Additionally, we excluded the data for one participant in ANT after additional pupil diameter visualization (cf. Figure 11) revealed the data was invalid. These modifications enhanced the effect size overall but did not affect the main results. • To stay within the word limit for the Introduction, we deleted some redundant sentences (lines 71-73 and 105-113). Also, some explanations in the Discussion (lines 482-491, 495-497, 503-504, 535-541, 584-599, 607-610) were deleted to avoid duplication. Additionally, the English editing service provider refined the text again, mainly in the discussion section (e.g., lines 442, 457-462, 505-520). • We changed one reference because we have found a study that deals with a frequency band more similar to the 10-second pupil trend (lines 470-475). • The "Pupil Measurement" section has been moved to the end of the Materials and Methods section to improve the flow of the manuscript. This is not highlighted because it does not involve the deletion of any existing text or the insertion of any new text.

Revised manuscript with deletions and insertions highlighted Abstract Maintaining concentration for several seconds on demanding cognitive tasks, like such as vigilance (VG) and working memory (WM) tasks, can lead to success or failure is crucial for successful task completion. Previous research has indicated suggests that internal concentration maintenance, known as tonic alertness, might be fundamental to the fluctuates, potentially declining to sub-optimal states, which can influence trial-by-trial performance across VG and WM in these tasks. However, the critical timescale of such alertness maintenance, manifested in the tonic signal of as indicated by slow changes in pupil diameter, has not been thoroughly investigated. This study investigated explored whether the "pupil trends," -which selectively reflecting signal sub-optimal tonic alertness maintenance at different various timescales-, exhibit a negatively linear relationship correlate with trial-by-trial performance in VG and WM tasks. InUsing the Psychomotor Vigilance Task (i.e., VG) and the Visual-Spatial 2-back Task (i.e., WM), we observed found that the human pupil trends at a lasting over 10- seconds timescale was were significantly higher in trials with longer reaction times, indicating poorer performance, compared to those with shorter reaction times trials, which indicateding better performance. Through the Attention Network Test, we further verified validated that thisese slow trends index specifically represents reflect sub-optimal states related to (tonic) alertness maintenance rather than another possible internal state associated with VG and WM performance, phasic alertness sub-optimal performance specific to VG and WM tasks, which is more associated with (phasic) responses to instantaneous interference. These findings underscore highlight the potential role of 10-second tonic alertness in VG and WM task performance variation, detecting and compensating for non-optimal states in VG and WM performance, significantly beyond the 10-second timescale. Additionally, the findings suggest suggesting the possibility of estimating human concentration during various visual tasks, even in the presence of when rapid pupil changes in response occur due to luminance fluctuations in various visual tasks.

Significance Statement Using biomarkers to estimate human concentration levels can adaptively enhance performance in daily activities. Theoretically, the pupil diameter that, which measurably fluctuates over several seconds, could mirror real-time concentration in demanding tasks like vigilance (VG) and working memory (WM). Although capable of accurately estimating concentration in the presence of rapid luminance changes, empirical evidence linking these pupil measures at the slow timescales to trial-by-trial VG and WM task performance of VG and WM tasks is lacking. This study demonstrates that the 10-second pupil trend accurately reflects these tasks' performance in these tasks, underscoring its potential for daily concentration assessment.

Introduction Success in maintaining concentration for several seconds varies, with failures potentially leading to significant consequences in for everyday activities. Cognitive psychology identifies vigilance (VG) and working memory (WM) tasks as requiring such concentration, with both showing trial-by-trial performance variations (DeBettencourt et al., 2019). A VG task is an attention task that demands readiness to respond to unpredictable stimuli within a 2 to 10-second latency period, exemplified by the Psychomotor Vigilance Task (PVT; Loh et al., 2004; Wilkinson &Houghton, 1982). WM tasks involve retaining a stimulus while processing another stimulus, requiring retrieval after a period exceeding 5 seconds, such as in the 2-back task (2BT; Carlson et al., 1998; Cohen et al., 1997). Since tThe prolongedmaximum variable latency period of (up to 10- seconds) is a critical commonality between VG and WM tasks (Unsworth et al., 2020; Unsworth &Robison, 2020). Therefore, shared variations fluctuations in internal state 2 maintenance at the critical relatively long timescales (e.g., 10 seconds) may underlie explain trial-by-trial performance variations in these tasks.

PupillometricNeuroscience studies have suggested that such variations in internal state maintenance may be reflected in the slow temporal component of pupil changes may reflect such variations in internal state maintenance (see Table 1; Aston-Jones &Cohen, 2005; Unsworth &Robison, 2017). Meta-analyses have identified The neural networks commonly active during involving VG and WM tasks like-such as the thalamus, anterior insula, and frontal operculum (AI/FO; Langner &Eickhoff, 2013; Rottschy et al., 2012)-. These regions slowly detect and signal the need to internally maintain response preparation for unpredictably timed targets, with increased AI/FO activity indicating poorer performance (i.e., "tonic" alertness" maintenance;

Coste &Kleinschmidt, 2016; Sadaghiani &D'Esposito, 2015; Sterzer &Kleinschmidt, 2010; Sturm &Willmes, 2001). The AI/FO activity increases with insufficient alertness (poorer performance) as the need for maintenance increases due to persistent adverse effects (Sadaghiani &D'Esposito, 2015). Through Tthe locus coeruleus-norepinephrine (LC-NE) system's anatomical ties with the pupil and AI/FO connected to pupil diameter regulation, mean that signals of internal maintenance inefficiency common to VG and WM tasks (i.e., suboptimal tonic alertness) may appear would manifest in the slow temporal component of the pupil changes, which negatively correlates with trial-by-trial task performance (Aston-Jones &Cohen, 2005; Gilzenrat et al., 2010; Joshi et al., 2016; Medford &Critchley, 2010; Schneider et al., 2016; Ullsperger et al., 2010). Moreover, tThese slow responses contrast with the an instantaneous statefast AI/FO (pupil) response to external interference events, which deviates from the preceding preparatory state, thereby indicating improved event detection performance (i.e., "phasic" alertness" response positively correlated with performance; Corbetta &Shulman, 2002;

3 Harsay et al., 2018; Menon &Uddin, 2010; Sterzer &Kleinschmidt, 2010; Tian et al., 2014; Ullsperger et al., 2010). Therefore, these tonic biomarkers may differ from the fast temporal component of the pupil changes, which positively correlates with performance (i.e., optimal phasic alertness).

HoweverDespite these backgrounds, previous analyses pupillometric studies have left a gap in evidence for the tonic pupil signals negatively related to trial-by-trial VG and WM performance (cf. Martin et al., 2022). Various studies have introduced task-dependent, not arbitrarily determined, temporal indices to capture the characteristics of pupil diameter, possibly lacking the temporal component that best reflects the tonic signal. For example, "Baseline (pretrial) pupil size" is thought to reflect internal states present before the start of a trial, potentially influencing responses after the variable latency period (e.g., 2 to 10 seconds) or retention period (e.g., 5 seconds; ) periods (Robison &Unsworth, 2019; Unsworth &Robison, 2018). However, findings regarding the relationship between baseline pupil size and task performance have been inconsistent. Studies have consistently shown a negative correlation between baseline variability and performance (Unsworth &Miller, 2021; Unsworth &Robison, 2017), but it remains unclear whether increases or decreases in baseline size are more closely linked to performance outcomes (Martin et al., 2022). This inconsistency likely arises from the wide range of timescales involved in the baseline pupil size, which may obscure the specific role of tonic pupil signals over long timescales that would negatively associate with performance (e.g., Van Den Brink et al., 2016). Capturing pupil changes in such a task-dependent and temporally variable manner may have prevented effective reflection of trial-by-trial performance leading to the only reflection of overall performance per individual (Unsworth &Miller, 2021; Unsworth &Robison, 2017).

To address these inconsistencies, this study introduces 'pupil trends' as novel indices that 4 capture slow, sustained changes in pupil diameter and explores long-timescale changes (e.g., 10 seconds) that negatively correlate with VG and WM performance. Therefore, this study examines how different temporal components of slow pupil changes negatively correlate with trial-by-trial VG and WM performance, providing convergent evidence for the pupil signal indicative of suboptimal tonic alertness (cf. Table 1). We introduce "pupil trends" as novel indices, differently capturing slow temporal changes (cf. Figure 1), iInspired by previous temporal analyses of pupil changes (Yamashita et al., 2021; 2022). Utilizing smoothing methods, we isolated pupil diameter changes at specific timescales using smoothing methods. This procedure filters out devoid of fast temporal components affected influenced by the rapid luminance fluctuations in tasks or the phasic alertness responses.

We examined the relationship between pupil trends with different temporal resolutions and trial-by-trial performance in PVT (i.e., VG) and 2BT (i.e., WM) tasks (Carlson et al., 1998; Cohen et al., 1997; Loh et al., 2004; Wilkinson &Houghton, 1982). Furthermore, we utilized employed the Attention Network Test (ANT; Fan et al., 2005) to demonstrate that the pupil trends signals the need for maintaining response readiness for unpredictable targets (tonic alertness ; Sadaghiani &D'Esposito, 2015) without indicating immediate interference detection or resolution (rather than the phasic alertness response to external interference). Given that theSince pupil responses to changes in stimulus luminance changes are largely instantaneous typically immediate, finding evidence that signals reflecting related to human concentration present persist over these slow timescales could be the first represent an essential step for toward estimating concentration in during complex cognitive tasks.

5 Materials and Methods Participants Part-time job applicants (N = 20, 15 females, age range, 20 and 43 years) with normal or corrected-to-normal vision participated in this study. The [Author University] Ethics Committee approved all experimental procedures. Written informed consent was obtained from every participant. They were recruited externally and compensated for participating.

Apparatus and Stimuli Participants were seated 60 cm from a 27-inch LCD monitor with a 144Hz refresh rate and 1920×1080 resolution. They engaged with stimuli presented on a black background via a Python (2.7.14) and Psychopy2 (Peirce, 2007; 2009) environment controlled by a desktop computer (Windows 7). Binocular eye movements were recorded at 1,000 Hz using the SR Research Eyelink 1000, with participants' heads stabilized by a chin-rest throughout the tasks.

Procedures We, in this study, aimed to provide convergent evidence for the that slow pupil signals indicative of insufficient (sub-optimal) tonic alertness potentially may underlieying the shared trial-by-trial performance decrements in VG and WM tasks. Participants performed eight tasks, with three selected for analysis in this study (i.e., VG task [PVT], WM task [2BT], and ANT). The task order was randomized, and each task lasted approximately 10 minutes. Sessions were designed such that two participants alternated between performing tasks and taking breaks, with the entire procedure spanning 180-210 minutes, including the preparations.

Tasks Procedures We usedemployed a VG task (i.e., PVT), aligning which is associated with the tonic alertness concept, and a WM task (i.e., 2BT), which correspondsing to fast phasic alertness 6 concerning interference detection and resolution, to gather substantial evidence for the tonic alertness's relevance across both domains (cf. Table 1). In general, VG and WM tasks require, participants may detect the need to maintain a preparatory state to counteract non-optimality during the latency (VG) or retention (WM) periods (tonic alertness maintenance; Langner &Eickhoff, 2013; Posner, 1978; Posner &Boies, 1971; Rottschy et al., 2012), while. At the same time, participants must also manage they may also handle interference by identifying external targets amidst competing internal states, such as mind wandering (VG) or concurrent interfering memories (WM; phasic alertness response; Burgess &Braver, 2010; Langner &Eickhoff, 2013; Levens &Phelps, 2010; Sridharan et al., 2008; Unsworth &Robison, 2016). To separately attribute these aspects to VG and WM tasks, we employed the PVT (Loh et al., 2004; Wilkinson &Houghton, 1982) and the 2BT (Carlson et al., 1998; Cohen et al., 1997). Should the If pupil trends commonly display consistently show correlations, it would broadly underscore the tonic alertness's role in across these domains.

For the VG task, wWe selected the PVT-based simple reaction task described by Mueller and Piper (2014) and Wilkinson and Houghton (1982) for the VG task. The PVT has been widely utilized to explore the overlap in cognitive demands between VG and WM tasks (Unsworth &Miller, 2021; Unsworth et al., 2020; Unsworth &Robison, 2017). The PVT demands a swift response to targets after a variable latency period, necessitating participants to sustain their response preparation throughout this interval (tonic alertness maintenance; Langner &Eickhoff, 2013; Posner, 1978; Posner &Boies, 1971). This task contrasts with other VG tasks, such as the Sustained Attention to Response or the Continuous Performance Task. These alternative tasks could complicate the execution of specific reactions due to the repetitive nature of other required responses, a phenomenon known as interference detection/resolution 7 (Robertson et al., 1997; Rosvold et al., 1956).

For the WM task, we selected the 2BT, following the methodology of a prior study (Carlson et al., 1998). In the 2BT (WM task), the participants perform the interfering task of remembering the stimulus presented in trial n for retrieval in trial n+2 while remembering the stimulus presented in trial n+1 for retrieval in another trial n+3. This task challenges participants to correctly detect and resolve the two interfering memory tasks, requiring continuous detection and resolution of interference (phasic alertness response; Burgess &Braver, 2010; Levens & Phelps, 2010). This demand for explicit detection and resolution of interference distinguishes the 2BT from tasks like the whole report procedure, which assesses memory capacity without directly addressing interference (Adam et al., 2015).

To examine the relationship between the pupil trend and individual performance in VG and WM tasks, we utilized reaction time (RT) as a differential marker of performance quality. An uptrend was predicted to correspond with poorer performance (longer RTs). RT in the PVT serves as a reliable VG performance indicator, varying from trial to trial (Unsworth et al., 2018). While WM performance in the 2BT is often gauged by accuracy (Haatveit et al., 2010), RT offered an alternative metric due to the scarcity of incorrect responses, thus providing a broader base for pupil index analysis. RT, within this context, is considered a proxy for WM performance (Jacola et al., 2014).

Further, utilizing the ANT (Fan et al., 2005) allowed us to examine whether the trend index signifiesd the need for tonic alertness maintenance for unpredictably timed targets rather than immediate external interference resolution, (phasic alertness response). The ANT's alerting comparison could differentiated between conditions with and without predictive cues for target timing,. This suggests positing that an absence of cues (no cue condition) would elevate the slow 8 pupil trend relative to conditions with the presence of cues (Coste &Kleinschmidt, 2016; Sadaghiani &D'Esposito, 2015; Sterzer &Kleinschmidt, 2010). Conversely, the executive control comparison, contrasting conditions with direct interference against those without (Geva et al., 2013; Laeng et al., 2011; Ullsperger et al., 2010), expected no significant variation in the pupil trend. We finally examined between-individual correlations in the effect sizes of pupil trends across PVT, 2BT, and ANT alerting comparisons, extending prior investigations that have highlighted shared performance dynamics in VG and WM tasks (Unsworth &Miller, 2021;

Unsworth et al., 2020; Unsworth &Robison, 2017).

PVT (VG) Methodology Participants were tasked with reacting to a target following a variable latency period ranging from 1,000 to 8,000 ms in 250 ms steps by pressing the "space" key as swiftly as possible (see Figure 21). This 1-8 seconds latency span bridges the common standard period of 2-10 seconds (Loh et al., 2004) and the shorter 1-4 seconds variant (Basner et al., 2011). The target, a white circle with a visual angle of 2.90 degrees, appeared at the screen's center. Each participant performed 29 latency periods, each consisting of four trials. Upon pressing the "space" key, the reaction time was shown for 1,000 ms. A "False Alarm!" alert was displayed if a participant responded before the target appeared. Conversely, failing to respond within 60 seconds triggered a "Miss!" alert. Following the disappearance of these alerts, the subsequent trial commenced immediately.

2BT (WM) Methodology In the 2BT, participants determined if the current target matched the position of the one presented two trials earlier. They responded by pressing the "right" (for "same") or "left" (for "different") key as promptly as possible (refer to Figure 32). A fixation point remained constant 9 at the screen's center, flanked by four rectangular frames. Each trial involved one frame turning white (indicating the target) for 500 ms before reverting to black for 3,000 ms. The rectangular frames set 1.48 visual degrees in size were positioned 3.80 degrees from the screen's center horizontally and vertically. Initially, each of the four positions had an equal (25%) chance of being the target. Subsequently, target selection was based on a 50% chance from locations used in the two preceding trials, combined with the remaining positions. A non-target position from the previous trials was chosen with a 33% probability for the latter. Participants encountered 160 targets across 158 trials (cf., no response for the first two targets).

ANT Methodology In the ANT, participants determined the direction (right or left) of the central arrow in target stimuli by pressing the "right" or "left" keys swiftly on the keyboard (see Figure 43). The task began with a fixation period of random length (400-1,600 ms), followed by a 100 ms cue presentation. Cue types included no cue, double cue, center cue, or spatial cue, succeeded by a 400 ms fixation before target appearance. Targets could be congruent, neutral, or incongruent, featuring a central arrow flanked by four others, positioned above or below the fixation point with equal probability (50%) on a trial-by-trial basis. The direction of the central arrow varied randomly (50% chance). Target stimuli, measuring a total of 3.08 visual degrees with each arrow or line spanning 0.55 degrees and a 0.06-degree separation between adjacent elements, remained visible until a response was made or for a maximum of 1,700 ms. Following a response, targets vanished, leading to a variable post-target fixation period (3,600 ms minus the initial fixation duration). Participants completed 96 trials in total. The alerting comparison contrasted the center and no cues, regardless of target type. Conversely, the executive control comparison assessed performance differences between congruent and incongruent targets, irrespective of cue type, to evaluate conflict resolution capabilities.

Pupil Measurement Procedures For this purpose, wWe measured task-independent pupil changes within a 5-15 second interval, calculated from the smoothed time series of pupil diameter across the same timescales, as universal indices of pupil temporal components (cf. Figure 1). We employed a smoothing technique to calculate this "smoothed trend," demonstrating its significant effect size with straightforward methods. We also utilized multiresolution decomposition to determine which timescale changes in pupil trends were significant (i.e., "decomposed trend"). To underscore the relevance of the 5-15-second interval Additionally, we also presented indicators for changes occurring within intervals of less than 5 seconds and beyond 15 seconds, which were not the subject of statistical tests.

Pupil Trend Calculation Preprocessing began with the exclusion of time series data related to pupilar diameter during eye closures (see left panels of Figures 4 and 5). A noise-robust method identified blinking times (Hershman et al., 2018), with 200 ms before and after detected blinks excluded to avoid artifacts. Missing pupil diameter data were interpolated linearly.

The pupil trend, defined as the change in pupil diameter centered at the target presentation across all tasks and trials, was then calculated (refer tosee right panels of Figures 14 and 5). This calculation involved transforming the preprocessed pupil diameter data into trend components through smoothing or multiresolution decomposition. The changes in these rigorously addressing the possibility of luminance difference between double cue and no cue affecting the biological signal (here, pupil signal), following previous studies (Fan et al., 2005). Also, this study did not include the comparison of orienting effects based on spatial versus center cues.

11 smoothed or decomposed diameters was were calculated, by averaging the indices for both eyes to account for binocular measurements.

Smoothed Trend For smoothing, we initially processed the time series of pupil diameter time series using smoothing windows of 205, 1510, 10, and 15 seconds (see Figure 4). This procedure involved calculating average values across these windows as they moved through the preprocessed time series of blink-interpolated pupil diameters data. Subsequently, trend indices were derived from the variations changes in pupil diameter corresponding to the width of over the respective windows durations.

For example, with a 210-second window, the change in pupil diameter across over this period (20 seconds) was quantified by measuring to determine the extent of increase or decrease. The change was calculated by subtracting the smoothed pupil diameter at the beginning of the 10-second window from that at the end. The value at the start represents the smoothed pupil diameter 5 seconds before the target presentation (i.e., the average of the pre-smoothed pupil diameter from -10 to 0 seconds), and the value at the end represents the smoothed pupil diameter 5 seconds after the target presentation (i.e., the average of the pre-smoothed pupil diameter from 0 to 10 seconds).

As a supplementary measure, we used the "conservative trend," which calculates changes over 14-second intervals while using a 10-second window for smoothing. In this approach, the initial smoothed value is the pupil diameter 7 seconds before the target presentation (i.e., the mean of the raw pupil diameter from -12 to -2 seconds), and the final smoothed value is the pupil diameter 7 seconds after the target presentation (i.e., the mean of the raw pupil diameter from 2 to 12 seconds). This conservative analysis was designed to carefully 12 exclude the effects of phasic activity that might occur within a 4-second range around the target presentation.

Decomposed Trend In the multiresolution decomposition approach, we segmented the pupil diameter time series data of pupil diameter into components of with varying temporal resolutions (see Figure 5). This segmentation was accomplished using a discrete wavelet transform, specifically the Symlet-4 wavelet (Lee et al., 2019). This technique allowed for scalings at resolutions of 2n, including 66 (65,536 ms), 33 (32,768 ms), 16 (16,384 ms), 8 (8,192 ms), 4 (4,096 ms), down to 0.002 (2 ms) seconds.

Each scaling level, starting from the 66-second scale, contained data on the absolute pupil diameter value and the magnitude of change in pupil diameter appropriate to at that specific temporal scale. Signals at the 33- seconds scale and below were distinct, focusing solely on the magnitude of changes at those finer resolutions, isolated from the broader- scale changes fluctuations. This procedure enabled us to calculate the pupil diameter variance precisely by analyzing the data obtained from each distinct temporal scale, specifically at the 33-, 16-, 8-, and 4-second resolutions.

For instance, at an 8-second resolution, the change in pupil diameter over this period was quantified. By examining the time series data at an 8-second temporal resolution, we compared the pupil diameters 4 seconds before and after the target presentation. The trend was calculated by subtracting the earlier value from the later one.

Code availability The data analysis code/software is freely available online at [URL redacted for double- blind review]. Researchers can use the code under a license set by [author's organization], which 13 allows them to use it for scientific evaluation. The data analysis was performed in a Python (3.8.17) environment controlled by a laptop computer (Windows 10, Intel Core i5-8265U CPU, 8GB RAM).

Results In the data analysis, we first presented the trial-by-trial differences in pupil trends between varying VG and WM performances in a straightforward manner (i.e., preliminary analysis) before examining which specific temporal resolutions of the trends differed between distinctly different performance levels (i.e., primary analysis).

Descriptive Statistics PVT (VG) Valid pupil data were provided by eighteen 18 participants after excluding one due to technical issues and another for having their eyelids partially closed throughout the task. These participants conducted a total of 2,088 trials. They accurately responded to most trials, with no missed trials and only seven nine false alarms, including anticipatory reactions with RTs of 150 ms or less. Erroneous trials were excluded from the analysis. The initial 5% (six trials) were also removed from analysis to eliminate potential noise from task initiation, such as pupil diameter adjustments to darkness, leaving 1,9731 of the 2,088 trials valid. The average reaction time (RT) for each participant for these trials was 399.13390.31 ms, with an average standard deviation (SD) of 108.7887.94 ms.

2BT (WM) SeventeenPparticipants (N =17) contributed valid pupil data after excluding one for technical errors, another for consistent partial eyelid closure, and a third for uncorrected vision during the task because the participants forgot to wear their eyeglasses. They completed a total of 14 2,686 trials. Of these, 2,4222,435 trials were answered correctly, with 2439 incorrect answers, 152 instances of no response, and no premature reactions under 150 ms. RT in 2BT aimed not just to measure reaction speed but also to serve as an alternative indicator of WM performance due to the variability in correct responses (mean incorrect responses were only 15.53). Only Incorrect- and non-responses were excluded. Additionally, the first 5% (eight trials) were omitted, resulting in 2,5382,313 valid trials out of 2,686. The average RT for each participant was 1,130.441,098.15 ms, with an average SD of 408.36376.67 ms.

ANT EighteenThe 17 participants yielded valid pupil data after excluding one for technical errors and two whose eyelids were half-closed throughout the task. These participants completed 1,7281,632 trials, accurately responding to 1,657 1,551 trials, with 58 incorrect responses, 1323 instances of no reaction, and no premature responses within 150 ms or less. Excluding erroneous trials and the initial 5% for valid conditions (eightthree trials) for potential start-up noise, 1,57003 out of 1,728632 trials were deemed valid. TheEach participant's average reaction time (RT) for each participant was 640.79673.72 ms, with an average standard deviation (SD) of 111.57119.99 ms.

Comparison of Pupil Trend Between PVT (VG) and 2BT (WM) PerformancePreliminary Analysis We first examined a the correlation between the smoothed pupil trend and performance timing in PVT (VG) and 2BT (WM), categorizing trials with RTs below the participant's average as good performance and those with longer RTs as poor. An Aanalysis was conducted to determine if average pupil trends varied between these performance levels in both tasks after excluding a few trials at the start and the end of the tasks without valid pupil trend data due to 15 smoothing window constraints. We also used the 'conservative trend' to highlight trend differences while excluding pupil changes around the target presentation. Additionally, Wwe also present conducted supplementary analyses comparing correct and incorrect responses in the 2BT.

For PVT (VG), the average RT for short response trials was 341.19 344.52 ms (SD = 32.17 32.69), while long response trials averaged 459.31 471.67 ms (SD = 76.16 89.68; t(17) = 8.11 -7.67, d = -1.96 - 1.83, p < .0001). In 2BT (WM), short RTs averaged 837.24 869.72 ms (SD = 160.63 162.08), with long RTs at 1,437.33 1490.30 ms (SD = 310.89 321.07; t(16) = -13.74 13.50, d = -2.35 -2.37, p < .0001). Additionally, the average RT for correct responses in 2BT was 1,096.46 1,098.15 ms (SD = 221.07 222.05), and for incorrect responses, 1,442.84 1439.05 ms (SD = 306.19 304.59; t(16) = -6.91 -6.80, d = -1.246, p < .0001).

Smoothing The initial analysis focused on pupil trends derived from moving averages, which revealed relatively large effect sizes for windows above 10 seconds. For PVT, the analysis of smoothed trends from various smoothing windows (Figure 56a) revealed that trends from a 10second windows above 5 seconds were significantly lower in short RT trials compared to than long RT trials ones (p < .05, False Discovery Rate [FDR] corrected, one-tailed). Similarly, in 2BT (Figure 6ba), trends from windows over ranging from 15 to 5 seconds showed significantly lower values in short RT trials compared to than in long RT trials ones (p < .05, FDR corrected).

Also, the 10-second conservative trends were lower in short RT trials than long ones (VG: t(17) = -3.67, d = -1.53, p < .001; WM: t(16) = -2.53, d = -1.17, p = 0.011). Additionally, a supplementary comparison of 2BT correct and incorrect responses (Figure 76c) indicated that trends over from 10- to 5-second windows were significantly lower in correct trials compared to incorrect ones (p < .05, FDR corrected). Although we did not develop a precise transformation 16 formula, it is worth noting that the 10-second trend differences (59.23 pixels in the PVT and 56.61 pixels in the 2BT) were approximately 0.07-0.08 mm. This estimation is based on the assumption that the mean pupil size at the PVT target presentation of 3,300 pixels corresponds to a generally observed pupil size of 4.5 mm against a dark background in a dimly lit room (Peysakhovich et al., 2015).

Multiresolution DecompositionPrimary Analysis We conducted a rigorous investigation to determine which temporal resolutions of the trends differed between markedly different performance levels.

PVT (VG) and 2BT (WM) We examined trend differences in PVT (VG) and 2BT (WM) by categorizing trials with reaction times (RTs) below the participant's 25th percentile as indicative of solid performance and those above the 75th percentile as indicative of poor performance. We first conducted the analysis using smoothed trends to ensure consistency with the preliminary analysis. We then applied decomposed trends to identify significant changes across different timescales.

For PVT (VG), the average RT for the fastest 25% of trials was 319.57 ms (SD = 28.01), while the slowest 75% averaged 498.26 ms (SD = 97.16; t(17) = -8.57, d = -2.43, p < .001). In 2BT (WM), the fastest 25% of short RTs averaged 723.07 ms (SD = 137.70), and the slowest 75% averaged 1,657.05 ms (SD = 367.19; t(16) = -13.79, d = -3.27, p < .001).

The initial analysis of smoothed trends across different temporal scales for PVT and 2BT, as illustrated in Figures 7 and 8, revealed that trends over 5-second windows in short RT trials were significantly lower than in long RT trials (p < .05, FDR corrected), with a slightly larger effect size observed for the 15-10-second window compared to adjacent windows. Further investigation analysis involved calculating independent pupil trends over these timescales 17 calculated through using multiresolution decomposition. The Aanalysis of decomposed trends across different temporal scales for PVT and 2BT, as shown in Figures 5b9 and 6b10, respectively, found that trends from 16- (orand 8-) second scales in short RT trials were significantly lower than in long RT trials (p < .05, uncorrected).

Comparison of Pupil Trend Between Different Conditions in the ANT Finally, Wwe then investigated whether the smoothed or decomposed trend index indicated reflected the necessity need for sustained response preparation maintenance in the face of temporally unpredictable targets, aligning consistent with the concept of tonic alertness slow internal non-optimality detection and compensation (Sadaghiani &D'Esposito, 2015) rather than phasic detectiong of deviations (such as stimulus interference in this study(phasic alertness).

In the alerting condition, the center-cue scenario, which externally indicated the timing of target presentation, contrasted with the no-cue condition that necessitated the participant's sustained alertness throughout the variable interval, potentially impacting the pupil trend. Mean reaction time (RT) for center-cue trials was 655.62 685.10 ms (SD = 90.87 144.37), compared to 677.68 714.88 ms (SD = 106.74 165.39) for no-cue trials, t(176) = -3.16 -3.50, d = -0.22 -0.19, p < .01.

Smoothing Analysis of ANT trends using different smoothing windows for the alerting condition (Figure 8a11) confirmed our expectations. , showing sSignificantly lower pupil trends were observed in center-cue trials compared to no-cue trials from in the 15- to 10-second windows (p < .05, FDR corrected). The 10-second trend difference was approximately 0.070 mm (51.13 pixels). The conservative trend for a 10-second window also showed a significant difference (t(16) = -2.64, d = -0.77, p < .009).

18 Multiresolution Decomposition In the multiresolution decomposition analysis, trends across at different scales in the ANT for both alerting and executive control comparisons (Figures 128b and 9b) showed that the trends from at the 16-second scales in center-cue trials wasere significantly lower than in no-cue trials (p < .05, uncorrected). However, there were no significant differences between congruent and incongruent target trials.

Conversely, the congruent target condition, lacking interference, differed from the incongruent target condition that entailed interference, a distinction we anticipated would not influence the pupil trend (i.e., executive control comparison). The mean RT for congruent targets was 628.93 667.12 ms (SD = 82.56 168.45), against 696.06 729.26 ms (SD = 96.13 160.34) for incongruent targets, t(176) = -7.73 -5.98, d = -0.73 -0.37, p < .0001. The executive control comparison trends (Figure 9a13) revealed no significant differences.Between-Individual Analysis of Trend Differences in PVT (VG), 2BT (WM), and ANT Alerting In an additional analysis, we investigated the between-individual correlations of effect sizes for trend differences across conditions in PVT (VG), 2BT (WM), and the ANT alerting comparison. Specifically, we assessed whether individuals demonstrating significant trend differences between conditions in one task similarly exhibited these differences in another task. This analysis was conducted on 16 participants who provided valid data across all tasks. We focused on the effect sizes of trends from the 10-second smoothing window across different tasks, employing the Pearson correlation coefficient and Biweight midcorrelation (bicor). The bicor, particularly advantageous for its robustness to outliers in small sample sizes (Wilcox, 1997), helped in this evaluation. Figure 10 presents the results of these between-individual correlations. While no correlation was observed between the trends in PVT and ANT alerting conditions, a significant 19 positive correlation emerged between PVT and 2BT, as well as between 2BT and ANT alerting, according to the bicor findings.

Discussion The pupil trends, identified at an approximately 10-second timescales longer than 10 seconds, and indicative of sub-optimal tonic alertness maintenance, showed a positive (negative) correlation with trial-by-trial RTs (performance)(i.e., a negative correlation with performance) in the PVT and the 2BT. In the ANT, this trend differentiated conditions with and without a timing cue, distinguishing between efficient (optimal) and inefficient (sub-optimal) tonic alertness maintenance. Furthermore, the presence or absence of target interference did not influence the trend, which indicates no reflection of indicating that it is unrelated to the phasic alertness response. Our findings convergently support the notion that pupil-linked variations in suboptimal tonic alertness maintenance over a 10- seconds timescale relate contribute to the observed performance variations decrements in VG and WM tasks.

Earlier research has thoroughly examined fast pupil changes over a trial, i.e., the serial flow of the start of a trial, the latency or retention period, the target presentation, and a response (see Table 2). Studies have shown that "pupil dilations" (i.e., high temporal resolution changes during the latency or retention period) and "phasic responses" time-locked to the target presentation are positively correlated with trial-by-trial performance in VG, WM, and other tasks (e.g., Hood et al., 2022; Unsworth et al., 2020). However, this research did not demonstrate the slow pupil changes that might negatively correlate with performance. Earlier research provided pupillometric evidence of phasic alertness affecting trial-by-trial performance in VG, and WM tasks but fell short of demonstrateing the role of tonic alertness spanning 10 seconds. Previous Wwithin-individual correlational analyses previously using 'pretrial (baseline) pupil size' (i.e., 20 the absolute magnitude at the start of a trial) highlighted a gap in evidence for tonic pupil signals linearly related to trial-by-trial VG performance (cf. Introduction; Martin et al., 2022). SuchMost studies predominantly relied on pupil indices derived at from high temporal resolutions, potentially encapsulating capturing a wide array range of internal states, such as including tonic alertness maintenance at across different timescales, phasic alertness changes, and various other emotional aspects factors (Bradley et al., 2008; Robison &Unsworth, 2019; Unsworth & Robison, 2018; Unsworth et al., 2018).

On the contrary, our investigation employed pupil trends at lower temporal resolutions, likely isolating the signals of sub-optimal tonic alertness maintenance specific to over timescales greater than a 10- seconds timeframe. This methodological approach enabled allowed the pupil trend to emerge as a distinct biomarker of sub-optimal tonic alertness maintenance, especially in cases where alertness regulation fails to return to an optimal level despite continuous effort for more than 10 seconds, as seen with in VG and WM tasks. The current Our results align may also be in agreement with those of Van Den Brink et al. (2016), which indicated that increased pupil changes over 30 seconds correlateds with reduced VG performance. The effectiveness of the slow trend calculations may also shed light on the neural basis of resting-state pupil fluctuations below 0.1 Hz, which are associated with increased fatigue or drowsiness (Wilhelm et al., 1998). be consistent with the notion that the degree of change in pupil diameter, rather than its absolute magnitude, more accurately mirrors the activity of the LC-NE system, distinguishing it from the influences of other neural systems, such as the acetylcholinergic system (Reimer et al., 2016). Furthermore, Oour novel index is robust against rapid luminance fluctuations, which is a desired characteristic making it suitable for assessing concentration maintenance across diverse various real-world tasks in under different luminance scenarios lighting conditions.

21 Complementary Pupillometric Approaches for Assessing Tonic and Phasic Alertness WhileAlthough the traditional pupil indices (e.g., pretrial pupil size, pupil dilations, and phasic pupil responses) capture a wide broad range of internal states, they may not effectively distinguish differentiate the specific temporal aspects of tonic alertness maintenance. Previous studies have relied on baseline pupil sizes (pre-trial pupil size), measured before trial initiation, to assess tonic alertness (Robison &Unsworth, 2019; Unsworth &Robison, 2018). Also, researchers have examined how the diameter changes between two timings, such as the trial initiation and the target presentation, and the target presentation and a few seconds after the presentation, to examine phasic alertness (Robison &Unsworth, 2019; Unsworth &Robison, 2018; Unsworth et al., 2018). Although internal maintenance inefficiency (suboptimal tonic alertness) could vary at the critical timescale of 10 seconds, these indices were designed to capture the task-dependent, not the arbitrarily determined, temporal components, lacking a specific index of the 10-second state changes.

These conventional indices could support integrative theories, such as the adaptive gain theory LC-NE account of attention control and working memory (AGT; Aston-Jones &Cohen, 2005; Unsworth &Robison, 2017), by elucidating the function of between-individual differences. This framework posits that variations in tonic and phasic alertness within individuals correlate with changes in attentional and memory performance within the same individuals. Specifically, it has been observed that individuals exhibiting significant within-individual variations in tonic alertness (pretrial pupil size) tend to have lower phasic alertness (pupil) response and poorer performance on average in between-individual analyses. The conventional index's ability to capture broad performance variations across participants sufficed for betweenindividual differences analyses (Unsworth &Miller, 2021). However, accurately capturing the 22 nuanced details of within-individual tonic alertness fluctuations for different temporal resolutions may necessitate a more refined approach and an index with a precise temporal resolution that can maximize the effect size for detecting subtle performance variations within an individual.

Introducing the pupil trend metric has offers the advantage of capturing the detailed characteristics of slow changes in internal states, such as specifically different timescales of tonic alertness fluctuations. In our approach, differences in the low-resolution pupil index, in the absence of without corresponding differences in the high-resolution differences, index indicate suggest slow tonic alertness fluctuations after excluding fast pupil changes caused by various factors. Thise present study exemplifies this approach, demonstrates how isolating sub-optimal tonic alertness maintenance fluctuations over a 10- seconds period, thereby helps elucidate filling the void in evidence supporting AGT, particularly regarding trial-by-trial tonic alertness regulation within individual performance across key VG and WM tasksparadigms.

ConventionallyTraditionally, tonic alertness is presumed has been assumed to precede occur prior to trial initiation, and is inferred from pre-trial pupil diameter measurements. This approach, however, may limit the detection of tonic alertness detection to the period before the latency or retention periods phases, potentially overlooking its active presence closer to the target presentation. The findings from this study suggest that variations in tonic alertness maintenance are not merely passive phenomena occurring well before target onset. but, Iinstead, they actively manifest around the time of the target processingpresentation.

Possible Mechanisms of Internal Alertness State Regulation: Proactive Versus Reactive Adjustments Given tThe roles of the AI/FO, LC-NE, and thalamus, offer insights into the observed pupillometric patterns likely reflect the regulation of intrinsic alertness to counteract decreased 23 tonic alertness caused by internal (PVT and 2BT) or external (ANT) factors. Overall (tonic) alertness affecting performance can be influenced by three factors: internal voluntary (i.e., intrinsic alertness), internal involuntary (e.g., fatigue), and external (e.g., uncertainty in target presentation) factors (Sturm &Willmes, 2001). These neural structures may engage regulate intrinsic alertness in two types of regulatory processes: proactive and reactive, facilitating helping to transitions from non-suboptimal to optimal states for task performance execution (Corbetta &Shulman, 2002; Menon &Uddin, 2010; Sadaghiani &D'Esposito, 2015; Sterzer &Kleinschmidt, 2010; Tian et al., 2014).

In this transition, the AI/FO may detect internal involuntary non-optimality,Suboptimal conditions might include such as cognitive fatigue (CF; Anderson et al., 2019), or default mode network (DMN; Sridharan et al., 2008) activation (Ullsperger et al., 2010), or even a taskoriented state in the presence of a task-irrelevant but important signal (Corbetta &Shulman, 2002). Activity within the AI/FO, LC-NE, and thalamus may be crucial for modulating these states to enhance task performance.

Proactive regulation involves the AI/FO's detection of slowly evolving suboptimal states independent of external stimuli, encompassing internally generated states related to CF or DMN activation (Ullsperger et al., 2010). The AI/FO's capability to identify It may also detect external non-optimality, such as the necessity for, and the detrimental impact on, maintaining response readiness during unpredictable intervals illustrates its sensitivity to tonic alertness inefficiencies not triggered by explicit stimulus presentation (Sadaghiani &D'Esposito, 2015). Detection Ssignals from the AI/FO, monitored by higher cognitive functions such as those in the prefrontal cortex, may gradually enhance intrinsic alertness to mitigate these adverse effects adjust these states towards optimal arousal and attention through the regulatory influence of the LC-NE and 24 thalamus (Langner &Eickhoff, 2013; Sadaghiani &D'Esposito, 2015).

When a participant's overall alertness is significantly reduced due to internal or external factors, restoring intrinsic alertness to an optimal state may take 10 seconds or more (i.e., suboptimal alertness maintenance). This finding could explain the 10-second pupil increase associated with below-average performance. The tonic signals lasting over 10- seconds tonic signal, identified in this study, potentially marks may reflect the proactive regulation of intrinsic alertness in response to non-suboptimal states factors during in VG and WM tasks. Variations in CF and DMN activity, possibly potentially mirrored in AI/FO activity (Anderson et al., 2019; Langner &Eickhoff, 2013; Sterzer &Kleinschmidt, 2010), may could influence compensatory pupil trends, that correlateing with RTs within PVT (VG) and 2BT (WM) tasks. When executingIn complex tasks such as like VG and WM tasks, higher-order functions intrinsic alertness regulation mightmay not fully compensate even if the AI/FO detected for nonsuboptimality tonic alertness detected by the AI/FO due to involuntary or external factors, resulting in leading to a negative relationship between the pupil trends (i.e., detected degree of suboptimal tonic alertness) and task performance.

Furthermore, the AI/FO's role in distinguishing the need for maintaining response preparation readiness under in the no- cue condition but not in the center -cue condition in of the ANT may result explain the observed differences in the pupil trends difference between thesem conditions (cf. Sadaghiani &D'Esposito, 2015). In the ANT, when a no-cue trial appears amid repeated trials indicating target presentation timing, alertness may drop significantly below the (increased) level required for task performance. Since this alertness deficit is not instantaneous, as in interference resolution, but persistent (tonic), intrinsic alertness regulation lasting more than 10 seconds may be activated after the perception of cue absence (cf. Sadaghiani &D'Esposito, 25 2015).

This tonic alertness regulation, occurring over 10 seconds (i.e., across several trials), may establish the prerequisite baseline for alertness regulation within shorter periods (i.e., a single trial; see Table 2). In the non-optimal range, the process of recruiting alertness may typically take 10-15 seconds, after which intermediate and phasic regulation can be effective. With the alertness around the optimal range, intermediate regulation may help transition overall alertness from slightly sub-optimal to optimal, focusing on target presentation timing. These different roles of tonic and intermediate alertness regulation may explain the observed correlations: baseline adjustment (pupil trend) negatively correlates with performance, while intermediate adjustment (pupil dilation) positively correlates with performance. These discussions may also contribute to a better understanding of attention and working memory, accounting for how significant within-individual tonic alertness variations lead to poorer overall performance per individual (Unsworth &Robison, 2017).

This discussion posits that the 10-second pupil trend reflects the intensity of detected suboptimal tonic alertness rather than the suboptimal state itself. This assumption aligns with the well-documented cognitive effort reflection by pupil dynamics (Kahneman, 1973; van der Wel &van Steenbergen, 2018) and is further elucidated by the AGT incorporating the Yerkes-Dodson's law (Aston-Jones &Cohen, 2005; Yerkes &Dodson, 1908). AGT, in harmony with Yerkes- Dodson's law, suggests optimal performance is achieved at an intermediate tonic alertness level (Aston-Jones &Cohen, 2005; Aston-Jones et al., 1999). Yerkes-Dodson's law also suggests that the maximal point of the inverted U-shaped curve varies depending on the task difficulty (Yerkes &Dodson, 1908). In other words, a higher (lower) task difficulty biases the maximal point toward smaller (larger) values, resulting in a negative (positive) linear relationship between tonic 26 alertness and task performance. In contrast, in the case of less challenging tasks, higher-order functions may fully compensate or overcompensate when the AI/FO detects suboptimal tonic alertness indicated by a positive relationship between the pupil trend (detected degree of suboptimal tonic alertness) and task performance. Our preliminary results suggest such a positive correlation in less challenging GO-NOGO tasks. Future studies are expected to support this hypothesis, providing tonic signal evidence for the AGT incorporating Yerkes-Dodson's law.

Moreover, during reactive after such regulation, the AI/FO -related networks are is known to instantaneously detect externally evoked interference (i.e., phasic response) (Corbetta &Shulman, 2002; Eckert et al., 2009; Menon &Uddin, 2010; Sridharan et al., 2008; Tian et al., 2014). This interference encompasses conflicts that are synchronized with the target onset, such as a more or less unexpected (salient) target appearance against the during a continuous preparatory state (Corbetta &Shulman, 2002), task-relevant targets emerging amid other internal activities like other task engagements or DMN activation (Menon &Uddin, 2010; Sridharan et al., 2008), and or targets surrounded by distracting stimuli (Eckert et al., 2009). In most experimental settings, accurately detecting such interference correlates with better task performance, implying that heightened phasic alertness, characterized by immediate pupil response to target presentation, enhances performance (Robison &Unsworth, 2019; Unsworth et al., 2018). The pupil trend differs from pupil changes reflecting phasic responses due to its temporal resolution and because it showed no effect in the executive control comparison in the ANT. Since this remains speculative, future studies are needed to fully capture the temporal dynamics of alertness regulation by analyzing tonic (pupil trend), intermediate (pupil dilation), and phasic (phasic pupil response) changes, thus covering the spectrum of internal nonoptimality, sub-optimality, and optimality.

27 Limitations and Future Directions This study is primarily an exploratory study with a small sample size, necessitating confirmatory analyses to validate the current hypotheses using a larger sample. We speculate that the primary effects (e.g., pupil trend differences over more than 10 seconds) should be replicable in future studies, as they generally showed sufficient statistical power in the current sample (d < 0.63, N = 17, 80% power, one-tailed). However, we could not investigate the exact scale at which the maximum effect occurs or the lower boundary at which the effect remains due to the limited statistical power (sample size) available for this exploratory study. Indeed, the nonsignificant effects in these ranges (-0.63 < d < 0) may become significant with a larger sample size.

Additionally, the various analyses conducted to ensure consistency and robustness (e.g., Steegen et al., 2016) may raise concerns about the familywise error rate due to multiple comparisons. This study corrected multiple comparisons for tests using strongly correlated variables (see FDR corrections for smoothed trends). From this perspective too, future studies are needed to confirm the current exploratory findings.

While currently speculative this study is currently exploratory, this discussion it highlights the need for further research to elucidate these mechanisms comprehensively. Integrating pupil trend analysis with brain imaging techniques could unravel the central and autonomic nervous systems' roles in modulating tonic and internal states of concentration during task execution. Moreover, the resilience of pupil trends to rapid luminance fluctuations might shed light on concentration dynamics in more naturalistic tasks, where stimulus luminance variations are less predictable.

28 Conclusion This study tested the hypothesis that variations in tonic alertness maintenance underlie shared trial-by-trial performance in VG and WM tasks. We demonstrated that the pupil trends over a lasting more than 10- seconds timescale, indicative of sub-optimal tonic alertness maintenance, is are negatively correlated with performance on a trial-by-trial basis in both VG and WM task types. Additionally, we provided convergent evidence that these is pupil trends reflects sub-optimal tonic alertness maintenance persistently implicitly induced rather than optimal phasic alertness instantaneously evoked responses triggered by the explicit external interference eventsstimulus onset. These exploratory findings support suggest the notion that 10second tonic intrinsic alertness over a 10-second timescale is a fundamental component critical factor influencing performance variations in VG and WM tasks.

References Adam KC, Mance I, Fukuda K, Vogel EK (2015) The contribution of attentional lapses to individual differences in visual working memory capacity. J Cogn Neurosci 27(8):1601-1616. https://doi.org/10.1162/jocn_a_00811 Anderson AJ, Ren P, Baran TM, Zhang Z, Lin F (2019) Insula and putamen centered functional connectivity networks reflect healthy agers' subjective experience of cognitive fatigue in multiple tasks. Cortex 119:428-440. https://doi.org/10.1016/j.cortex.2019.07.019 Aston-Jones G, Cohen JD (2005) An integrative theory of locus coeruleus-norepinephrine function: adaptive gain and optimal performance. Annu Rev Neurosci 28:403-450. https://doi.org/10.1146/annurev.neuro.28.061604.135709 Aston-Jones G, Rajkowski J, Cohen J (1999) Role of locus coeruleus in attention and behavioral flexibility. Biol Psychiatry 46(9):1309-1320. https://doi.org/10.1016/s0006- 29 3223(99)00140-7 Basner M., Mollicone D, Dinges DF (2011) Validity and sensitivity of a brief psychomotor vigilance test (PVT-B) to total and partial sleep deprivation. Acta Astronaut 69(11- 12):949-959. https://doi.org/10.1016/j.actaastro.2011.07.015 Bradley MM, Miccoli L, Escrig MA, Lang PJ (2008) The pupil as a measure of emotional arousal and autonomic activation. Psychophysiology 45(4):602-607. https://doi.org/10.1111/j.1469-8986.2008.00654.x Burgess GC, Braver TS (2010) Neural mechanisms of interference control in working memory: effects of interference expectancy and fluid intelligence. PLoS One 5(9):e12861. https://doi.org/10.1371/journal.pone.0012861 Carlson S, Martinkauppi S, Rämä P, Salli E, Korvenoja A, Aronen HJ (1998) Distribution of cortical activation during visuospatial n-back tasks as revealed by functional magnetic resonance imaging. Cereb Cortex 8(8):743-752. https://doi.org/10.1093/cercor/8.8.743 Cohen JD, Perlstein WM, Braver TS, Nystrom LE, Noll DC, Jonides J, Smith EE (1997) Temporal dynamics of brain activation during a working memory task. Nature 386(6625):604-608. https://doi.org/10.1038/386604a0 Corbetta M, Shulman GL (2002) Control of goal-directed and stimulus-driven attention in the brain. Nat Rev Neurosci 3(3):201-215. https://doi.org/10.1038/nrn755 Coste CP, Kleinschmidt A (2016) Cingulo-opercular network activity maintains alertness.

Neuroimage 128:264-272. https://doi.org/10.1016/j.neuroimage.2016.01.026 DeBettencourt MT, Keene PA, Awh E, Vogel EK (2019) Real-time triggering reveals concurrent lapses of attention and working memory. Nat Hum Behav 3(8):808-816. https://doi.org/10.1038/s41562-019-0606-6 30 Eckert MA, Menon V, Walczak A, Ahlstrom J, Denslow, Horwitz A, Dubno JR (2009) At the heart of the ventral attention system: the right anterior insula. Hum Brain Mapp 30(8):2530-2541. https://doi.org/10.1002/hbm.20688 Fan J, McCandliss BD, Fossella J, Flombaum JI, Posner MI (2005) The activation of attentional networks. Neuroimage 26(2):471-479. https://doi.org/10.1016/j.neuroimage.2005.02.004 Geva R, Zivan M, Warsha A, Olchik D (2013) Alerting, orienting or executive attention networks: differential patters of pupil dilations. Front Behav Neurosci 7:145. https://doi.org/10.3389/fnbeh.2013.00145 Gilzenrat MS, Nieuwenhuis S, Jepma M, Cohen JD (2010) Pupil diameter tracks changes in control state predicted by the adaptive gain theory of locus coeruleus function. Cogn Affect Behav Neurosci 10(2):252-269. https://doi.org/10.3758/CABN.10.2.252 Haatveit BC, Sundet K, Hugdahl K, Ueland T, Melle I, Andreassen OA (2010) The validity of d prime as a working memory index: results from the "Bergen n-back task." J Clin Exp Neuropsychol 32(8):871-880. https://doi.org/10.1080/13803391003596421 Harsay HA, Cohen MX, Spaan M, Weeda WD, Nieuwenhuis S, Ridderinkhof KR (2018) Error blindness and motivational significance: shifts in networks centering on anterior insula co-vary with error awareness and pupil dilation. Behav Brain Res 355:24-35. https://doi.org/10.1016/j.bbr.2017.10.030 Hershman R, Henik A, Cohen N (2018) A novel blink detection method based on pupillometry noise. Behav Res Methods 50:107-114. https://doi.org/10.1080/10.3758/s13428-017- 1008-1 Hood AV, Hart KM, Marchak FM, Hutchison, KA (2022) Patience is a virtue: Individual 31 differences in cue-evoked pupil responses under temporal certainty. Atten Percept Psychophys 84(4), 1286-1303. https://doi.org/10.3758/s13414-022-02482-7 Jacola LM, Willard VW, Ashford JM, Ogg RJ, Scoggins MA, Jones MM, Wu S, Conklin HM (2014) Clinical utility of the N-back task in functional neuroimaging studies of working memory. J Clin Exp Neuropsychol 36(8):875-886. https://doi.org/10.1080/13803395.2014.953039 Joshi S, Li Y, Kalwani RM, Gold JI (2016) Relationships between pupil diameter and neuronal activity in the locus coeruleus, colliculi, and cingulate cortex. Neuron 89(1):221-234. https://doi.org/10.1016/j.neuron.2015.11.028 Kahneman D (1973) Attention and Effort.

Englewood Cliffs, NJ: Prentice-Hall Laeng B, Ørbo M, Holmlund T, Miozzo M (2011) Pupillary stroop effects. Cogn Process 12:13- 21. https://doi.org/10.1007/s10339-010-0370-z Langner R, Eickhoff SB (2013) Sustaining attention to simple tasks: a meta-analytic review of the neural mechanisms of vigilant attention. Psychol Bull 139(4):870-900. https://doi.org/10.1037/a0030694 Lee G, Gommers R, Waselewski F, Wohlfahrt K, O'Leary A (2019) PyWavelets: A Python package for wavelet analysis. J Open Source Softw 4(36):1237. https://doi.org/10.21105/joss.01237 Levens SM, Phelps EA (2010) Insula and orbital frontal cortex activity underlying emotion interference resolution in working memory. J Cogn Neurosci 22(12):2790-2803. https://doi.org/10.1162/jocn.2010.21428 Loh S, Lamond N, Dorrian J, Roach G, Dawson D (2004) The validity of psychomotor vigilance tasks of less than 10-minute duration. Behav Res Methods, Instruments, &Computers 32 36:339-346. https://doi.org/10.3758/bf03195580 Martin JT, Whittaker AH, Johnston SJ (2022) Pupillometry and the vigilance decrement: Task- evoked but not baseline pupil measures reflect declining performance in visual vigilance tasks. Eur J Neurosci 55(3):778-799. https://doi.org/10.1111/ejn.15585 Medford N, Critchley HD (2010) Conjoint activity of anterior insular and anterior cingulate cortex: awareness and response. Brain Struct Funct 214:535-549. https://doi.org/10.1007/s00429-010-0265-x Menon V, Uddin LQ (2010) Saliency, switching, attention and control: a network model of insula function. Brain Struct Funct 214:655-667. https://doi.org/10.1007/s00429-010-0262-0 Mueller ST, Piper BJ (2014) The psychology experiment building language (PEBL) and PEBL test battery. J Neurosci Methods 222:250-259. https://doi.org/10.1016/j.jneumeth.2013.10.024 Reimer J, McGinley MJ, Liu Y, Rodenkirch C, Wang Q, McCormick DA, Tolias AS (2016) Pupil fluctuations track rapid changes in adrenergic and cholinergic activity in cortex. Nat Commun 7(1):13289. https://doi.org/10.1038/ncomms13289 Peirce JW (2007) PsychoPy-psychophysics software in Python. J Neurosci Methods 162(1- 2):8-13. https://doi.org/10.1016/j.jneumeth.2006.11.017 Peirce JW (2009) Generating stimuli for neuroscience using PsychoPy. Front Neuroinform 2:10. https://doi.org/10.3389/neuro.11.010.2008 Peysakhovich V, Causse M, Scannella S, Dehais F (2015) Frequency analysis of a task-evoked pupillary response: Luminance-independent measure of mental effort. Int J Psychophysiol 97(1), 30-37. https://doi.org/10.1016/j.ijpsycho.2015.04.019 Posner MI (1978) Chronometric explorations of mind. Hillsdale, NJ: Lawrence Erlbaum.

33 Posner MI, Boies SJ (1971) Components of attention. Psychol Rev 78(5):391-408. https://doi.org/10.1037/h0031333 Robertson IH, Manly T, Andrade J, Baddeley BT, Yiend J (1997) 'Oops!': performance correlates of everyday attentional failures in traumatic brain injured and normal subjects.

Neuropsychologia 35(6):747-758. https://doi.org/10.1016/S0028-3932(97)00015-8 Robison MK, Unsworth N (2019) Pupillometry tracks fluctuations in working memory performance. Atten Percept Psychophys 81:407-419. https://doi.org/10.3758/s13414- 018-1618-4 Rosvold HE, Mirsky AF, Sarason I, Bransome Jr ED, Beck LH (1956) A continuous performance test of brain damage. J Consult Psychol 20(5):343-350. https://doi.org/10.1037/h0043220 Rottschy C, Langner R, Dogan I, Reetz K, Laird AR, Schulz JB, Fox PT, Eickhoff SB (2012) Modelling neural correlates of working memory: a coordinate-based meta-analysis.

Neuroimage 60(1):830-846. https://doi.org/10.1016/j.neuroimage.2011.11.050 Sadaghiani S, D'Esposito M (2015) Functional characterization of the cingulo-opercular network in the maintenance of tonic alertness. Cereb cortex 25(9):2763-2773. https://doi.org/10.1093/cercor/bhu072 Schneider M, Hathway P, Leuchs L, Sämann PG, Czisch M, Spoormaker VI (2016) Spontaneous pupil dilations during the resting state are associated with activation of the salience network. Neuroimage 139:189-201. https://doi.org/10.1016/j.neuroimage.2016.06.011 Sridharan D, Levitin DJ, Menon V (2008) A critical role for the right fronto-insular cortex in switching between central-executive and default-mode networks. Proc Natl Acad Sci U S A 105(34):12569-12574. https://doi.org/10.1073/pnas.0800005105 34 Steegen S, Tuerlinckx F, Gelman A, Vanpaemel W (2016) Increasing transparency through a multiverse analysis. Perspectives on Psychological Science 11(5):702-712: https://doi.org/10.1177/17456916166586 Sterzer P, Kleinschmidt A (2010) Anterior insula activations in perceptual paradigms: often observed but barely understood. Brain Struct Funct 214(5-6):611-622. https://doi.org/10.1007/s00429-010-0252-2 Sturm W, Willmes K (2001) On the functional neuroanatomy of intrinsic and phasic alertness.

Neuroimage 14(1):S76-S84. https://doi.org/10.1006/nimg.2001.0839 Tian Y, Liang S, Yao D (2014) Attentional orienting and response inhibition: insights from spatial-temporal neuroimaging. Neurosci Bull 30:141-152. https://doi.org/10.1007/s12264-013-1372-5 Ullsperger M, Harsay HA, Wessel JR, Ridderinkhof KR (2010) Conscious perception of errors and its relation to the anterior insula. Brain Struct Funct 214:629-643. https://doi.org/10.1007/s00429-010-0261-1 Unsworth N, Miller AL (2021) Individual differences in the intensity and consistency of attention. Curr Dir Psychol Sci 30(5):391-400. https://doi.org/10.1177/09637214211030266 Unsworth N, Miller AL, Robison MK (2020) Individual differences in lapses of sustained attention: Ocolumetric indicators of intrinsic alertness. J Exp Psychol Hum Percept Perform 46(6):569-592. https://doi.org/10.1037/xhp0000734 Unsworth N, Robison MK (2016) Pupillary correlates of lapses of sustained attention. Cogn Affect Behav Neurosci 16:601-615. 10.3758/s13415-016-0417-4 Unsworth N, Robison MK (2017) A locus coeruleus-norepinephrine account of individual 35 differences in working memory capacity and attention control. Psychon Bull Rev 24:1282-1311. https://doi.org/10.3758/s13423-016-1220-5 Unsworth N, Robison MK (2018) Tracking working memory maintenance with pupillometry.

Atten Percept Psychophys 80:461-484. https://doi.org/10.3758/s13414-017-1455-x Unsworth N, Robison MK (2020) Working memory capacity and sustained attention: A cognitive-energetic perspective. J Exp Psychol Learn Mem Cogn 46(1):77-103. https://doi.org/10.1037/xlm0000712 Unsworth N, Robison MK, Miller AL (2018) Pupillary correlates of fluctuations in sustained attention. J Cogn Neurosci 30(9):1241-1253. https://doi.org/10.1162/jocn_a_01251 Van Den Brink RL, Murphy PR, Nieuwenhuis S (2016) Pupil diameter tracks lapses of attention.

PLoS One 11(10):e0165274. https://doi.org/10.1371/journal.pone.0165274 van der Wel P, van Steenbergen H (2018) Pupil dilation as an index of effort in cognitive control tasks: A review. Psychon Bull Rev 25:2005-2015. 10.3758/s13423-018-1432-y Wilhelm B, Wilhelm H, Lüdtke H, Streicher P, Adler M (1998) Pupillographic assessment of sleepiness in sleep-deprived healthy subjects. Sleep 21(3):258-265. https://doi.org/10.1093/sleep/21.3.258 Wilkinson RT, Houghton D (1982) Field test of arousal: a portable reaction timer with data storage. Hum Factors 24(4):487-493. https://doi.org/10.1177/001872088202400409 Wilcox RR (1997) Introduction to robust estimation and hypothesis testing. Boston, US:

Academic Press.

Yamashita J, Terashima H, Yoneya M, Maruya K, Koya H, Oishi H, Nakamura H, Kumada T (2021) Pupillary fluctuation amplitude before target presentation reflects short-term vigilance level in Psychomotor Vigilance Tasks. PLoS One 16(9):e0256953.

36 https://doi.org/10.1371/journal.pone.0256953 Yamashita J, Terashima H, Yoneya M, Maruya K, Oishi H, Kumada T (2022) Pupillary fluctuation amplitude preceding target presentation is linked to the variable foreperiod effect on reaction time in Psychomotor Vigilance Tasks. PLoS One 17(10):e0276205. https://doi.org/10.1371/journal.pone.0276205 Yerkes RM, Dodson JD (1908) The relation of strength of stimulus to rapidity of habit- formation. J Comp Neurol 18:459-482.

Legends Table 1. Background Summary of Tonic and Phasic Signals Biomarkers in Demanding VG and WM Tasks.

Tonic maintenance signal Phasic response signal Meaning Alertness maintenance at the (increased) level required by persistent adverse factors Alertness response to external instantaneous deviation (e.g., salience or interference) onset Timescale Long Short Correlation with performance Negative Positive 37 Table 2. Summary of Tonic and Phasic Pupil Indices in VG and WM Tasks.

Long-term (tonic) ⚫ Beyond a trial Intermediate ⚫ Within a trial Short-term (phasic) ⚫ Instantaneous Pupil size Pretrial (baseline) size at the start of a trial: Larger variations negatively correlated with performance (Not applicable) (Not applicable) Pupil changes Pupil trends across several trials:

Negatively correlated with performance Pupil dilations between the start of a trial and the target presentation:

Positively correlated with performance Phasic pupil responses around the target presentation:

Positively correlated with performance Figure 1. Conceptual Illustration of Pupil Trend Calculation.

This figure illustrates the process of calculating the pupil trend within a PVT, featuring a latency period against a black background and a target presentation highlighted by a white circle. It displays the raw pupil diameter as a thin gray line, affected by immediate changes in screen luminance. Similarly, the pupil diameter smoothed over a 0.1-second window-a common practice in conventional pupillometric studies-appears as a thick green line and is influenced by luminance changes. In contrast, the diameter smoothed over a 10-second window is depicted as a thick blue line, highlighting the method's sensitivity to slower changes in pupil size that are not affected by rapid shifts in luminance. The trend calculation, focused on the period surrounding 38 the second target presentation, determines the pupil diameter's expansion or contraction over the specified 10 seconds, centered on the moment of the target display. This calculation is visually represented by the vertical length of the blue arrow, indicating the pupil trend's quantitative value.

Figure 12. Sequence of Elements in a Psychomotor Vigilance Task (PVT) Trial.

Figure 23. Sequence of Elements in a 2-Back Task (2BT) Response.

39 Figure 34. Attention Network Test (ANT) Trial Sequence.

This figure details the sequence of cue presentations in an ANT trial. The "No cue" condition shows only the fixation cross. The "Double cue" condition presents two asterisks, indicating both potential target positions (above and below). The "Center cue" condition features an asterisk at the fixation cross's location, while the "Spatial cue" condition places an asterisk at the actual target location, either position (above or below), as a valid predictor of the target's position location. The appearance of each cue type is randomized, occurring with equal probability (25%) across trials. The three kinds of target stimuli were as follows:. The congruent target stimuli consisted of five arrows pointing in the same direction;. Tthe neutral target stimuli consisted of a central arrow and four flankers of line segments;. Tthe incongruent target stimuli consisted of a central arrow and four flankers of arrows pointing in opposite directions.

40 Figure 4. Conceptual Illustration of Smoothed Trend Calculation. This figure illustrates the process of calculating the smoothed trend within a PVT. The left panel displays the raw pupil diameter (gray line), including blinks, and the post-blink interpolation (black line). The presmoothed pupil diameter rapidly constricts when the white target appears, as indicated by the blue marker. In contrast, the right panels show pupil diameters smoothed over 5-15-second windows, highlighting the method's sensitivity to slower pupil changes unaffected by rapid shifts in luminance. The trend calculation reflects the pupil's expansion or contraction over the specified time window, centered on the moment of the target display. This is calculated by subtracting the diameter indicated by the green marker from that indicated by the purple marker. In the case of 10-second smoothing, this corresponds to subtracting the mean pre-smoothed diameters shown in the light green graph from those in the light purple graph.

41 Figure 5. Conceptual Illustration of Decomposed Trend Calculation. This figure illustrates the calculation process for the decomposed trend within a PVT. The left panel shows the raw and blink-interpolated pupil diameters as gray and black lines, respectively. These diameters rapidly constrict when the white target appears, as indicated by the blue marker. In contrast, the right panels display the decomposed pupil diameter, representing the fluctuating component with a time resolution of 4-16 seconds, which is unaffected by rapid luminance shifts. In each decomposition, the trend calculation subtracts the decomposed diameter indicated by the green marker from that indicated by the purple marker.

42 Figure 6. Preliminary Pupil Comparison Using the Smoothing Method. A, Short and long RT in PVT (VG). B, Short and long RT in 2BT (WM). C, Correct and incorrect responses in 2BT (WM).

43 Figure 5. Comparison of Pupil Trend Between Short and Long RT in PVT (VG) Using (a) Smoothing and (b) Multiresolution Decomposition Methods. Figure 6. Comparison of Pupil Trend Between Short and Long RT in 2BT (WM) Using (a) Smoothing and (b) Multiresolution Decomposition Methods. Figure 7. Comparison of Pupil Trend between Correct and Incorrect Responses in 2BT (WM) Using Smoothing Method.

Figure 7. Pupillary Comparison in PVT (VG). Smoothed trend (Top); smoothed diameter (Bottom); between short (Blue) and long reaction times (Orange). The top panels present boxand-whisker plots of the trend indices. The bottom panels display the smoothed pupil diameters that serve as the source of the trend indices (solid line: mean smoothed pupil diameter for 30 seconds; translucent area: standard error). For visualization purposes, the smoothed pupil diameter for each participant is adjusted to the average diameter across participants at the start time of trend calculation.

44 Figure 8. Pupillary Comparison in 2BT (WM). Smoothed trend (Top); smoothed diameter (Bottom); between short (Blue) and long RT (Orange). The top panels display box-and-whisker plots of the trend indices. The bottom panels show the smoothed pupil diameters that serve as the source for the trend indices (solid line: mean smoothed pupil diameter for 30 seconds; translucent area: standard error). For visualization purposes, each participant's smoothed pupil diameter is adjusted to the average diameter across participants at the start of the trend calculation.

45 Figure 9. Pupillary Comparison in PVT (VG). Decomposed trend (Top); decomposed diameter (Bottom); between short (Blue) and long RT (Orange). The top panels present box-andwhisker plots of the trend indices. The bottom panels display the decomposed pupil diameters that serve as the source for the trend indices (solid line: mean decomposed pupil diameter for 30 seconds; translucent area: standard error). For visualization purposes, the decomposed pupil diameter for each participant is adjusted to the average diameter across participants at the start of the trend calculation.

46 Figure 10. Pupillary Comparison in 2BT (WM). Decomposed trend (Top); decomposed diameter (Bottom); between short (Blue) and long RT (Orange). The top panels display box-andwhisker plots of the trend indices. The bottom panels show the decomposed pupil diameters that serve as the source of the trend indices (solid line: mean decomposed pupil diameter for 30 seconds; translucent area: standard error). For visualization purposes, each participant's decomposed pupil diameter is adjusted to the average diameter across participants at the start of the trend calculation.

47 Figure 11. Alerting Comparison of the Pupil in ANT. Smoothed trends (Top); smoothed diameters (Bottom). Center cue (Blue), no cue (Orange). The top panels present box-and-whisker plots of the trend indices. The bottom panels show the smoothed pupil diameters that serve as the source of the trend indices (solid line: mean smoothed pupil diameter for 30 seconds; translucent area: standard error). For visualization purposes, each participant's smoothed pupil diameter is adjusted to the average diameter across participants at the start of the trend calculation.

48 Figure 12. Alerting Pupil Comparison in ANT. Decomposed trends (Top); decomposed diameters (Bottom). Center cue (Blue), no cue (Orange). The top panels present box-and-whisker plots of the trend indices. The bottom panels show the decomposed pupil diameters that serve as the source of the trend indices (solid line: mean decomposed pupil diameter for 30 seconds; translucent area: standard error). For visualization purposes, each participant's decomposed pupil diameter is adjusted to the average diameter across participants at the start of the trend calculation.

49 Figure 13. Executive Control Comparison of Pupil Trends in ANT. Smoothed trends (Top); smoothed diameters (Bottom). Congruent target (Blue), incongruent target (Orange). The top panels present box-and-whisker plots of the trend indices. The bottom panels show the smoothed pupil diameters that serve as the source of the trend indices (solid line: mean smoothed pupil diameter for 30 seconds; translucent area: standard error). For visualization purposes, each participant's smoothed pupil diameter is adjusted to the average diameter across participants at the start of the trend calculation.

Figure 8. Alerting Comparison of Pupil Trends in ANT Using (a) Smoothing and (b) Multiresolution Decomposition Methods.

Figure 9. Executive Control Comparison of Pupil Trends in ANT Using (a) Smoothing and (b) Multiresolution Decomposition Methods.

50 995 Figure 10. Between-Individual Correlation of Trend Differences in PVT (VG), 2BT (WM), 996 and ANT Alerting.

51
